# First-line penpulimab (an anti-PD1 antibody) and anlotinib (an angiogenesis inhibitor) with nab-paclitaxel/gemcitabine (PAAG) in metastatic pancreatic cancer: a prospective, multicentre, biomolecular exploratory, phase II trial

**DOI:** 10.1038/s41392-024-01857-6

**Published:** 2024-06-07

**Authors:** Huizi Sha, Fan Tong, Jiayao Ni, Yi Sun, Yahui Zhu, Liang Qi, Xiaoqin Li, Wei Li, Yan Yang, Qing Gu, Xing Zhang, Xiaoxuan Wang, Chan Zhu, Dongsheng Chen, Baorui Liu, Juan Du

**Affiliations:** 1 The Comprehensive Cancer Center, Nanjing Drum Tower Hospital, Affiliated Hospital of Medical School, Nanjing University & Clinical Cancer Institute of Nanjing University, Nanjing, China; 2 The Comprehensive Cancer Center, Nanjing Drum Tower Hospital, Clinical College of Traditional Chinese and Western Medicine, Nanjing University of Chinese Medicine, Nanjing, China; 3 Department of Oncology, The Affiliated Hospital of Jiangsu University, Zhenjiang, China; 4 Department of Oncology, The First Affiliated Hospital of Soochow University, Suzhou, China; 5 Department of Oncology, The Affiliated Jiangning Hospital of Nanjing Medical University, Nanjing, China; 6 National Institute of Healthcare Data Science at Nanjing University, Nanjing, China; 7 State Key Laboratory of Neurology and Oncology Drug Development, Jiangsu Simcere Diagnostics Co., Ltd., Nanjing Simcere Medical Laboratory Science Co., Ltd., Nanjing, China

**Keywords:** Gastrointestinal cancer, Tumour immunology, Predictive markers, Tumour angiogenesis, Metastasis

## Abstract

Metastatic pancreatic cancer (mPC) has a dismal prognosis. Herein, we conducted a prospective, multicentre, single-arm, phase II trial evaluating the efficacy and safety of penpulimab and anlotinib in combination with nab-paclitaxel/gemcitabine (PAAG) in patients with first-line mPC (NCT05493995). The primary endpoints included the objective response rate (ORR) and disease control rate (DCR), while secondary endpoints encompassed progression-free survival (PFS), overall survival (OS), and safety. In 66 patients analysed for efficacy, the best response, indicated by the ORR, was recorded at 50.0% (33/66) (95% CI, 37.4–62.6%), with 33 patients achieving partial response (PR). Notably, the DCR was 95.5% (63/66, 95% CI, 87.3–99.1%). The median PFS (mPFS) and OS (mOS) were 8.8 (95% CI, 8.1–11.6), and 13.7 (95% CI, 12.4 to not reached) months, respectively. Grade 3/4 treatment-related adverse events (TRAEs) were reported in 39.4% of patients (26/66). In prespecified exploratory analysis, patients with altered SWI/SNF complex had a poorer PFS. Additionally, low serum CA724 level, high T-cell recruitment, low Th17 cell recruitment, and high NK CD56dim cell scores at baseline were potential predicative biomarkers for more favourable efficacy. In conclusion, PAAG as a first-line therapy demonstrated tolerability with promising clinical efficacy for mPC. The biomolecular findings identified in this study possess the potential to guide the precise clinical application of the triple-combo regimen.

## Introduction

Pancreatic cancer (PC), a common malignant tumor of the digestive system, has shown a gradual increase in incidence and mortality worldwide. It is estimated that PC will become the second leading cause of cancer-related deaths worldwide by the year 2030.^1^ Currently, systemic chemotherapy remains the cornerstone of treatment for advanced metastatic PC (mPC). Both the AG regimen (nab-paclitaxel/gemcitabine) and FOLFIRINOX regimen (oxaliplatin + irinotecan + calcium folinate + 5-FU) have objective response rates (ORR) of approximately 20%, and overall survival (OS) does not exceed one year.^2,3^ While immune checkpoint inhibitors (ICIs) have transformed cancer treatment in the last decade, their clinical benefits in PC remain to be substantiated. Both the CISPD3 study^4^ (NCT03977272) and the PRINCE study^5^ (NCT03214250) suggest that the combination of chemotherapy and ICI in PC can improve the ORR, reaching ~50%. This indicates that the combination of immunotherapy and chemotherapy has certain potential clinical significance. However, the key challenge for future research lies in translating the ORR benefit into improvements in OS and progression-free survival (PFS).

The interaction between the activation of angiogenic signalling pathways and the suppression of tumor immunity has been validated. Remarkably, antiangiogenic drugs, through direct inhibition of tumor growth and metastasis, can reprogram the tumor microenvironment from an immunosuppressive state to permissiveness.^6^ For instance, patients with advanced intrahepatic cholangiocarcinoma (ICC) received a combination of toripalimab, lenvatinib, and gemcitabine plus oxaliplatin (GEMOX) (NCT03951597), reaching a breakthrough ORR of 80%.^7^ The DRAGON IV study (NCT04208347) also confirmed the safety and a significantly higher pathologic complete response (pCR) rate than standard care (18.3% vs. 5.0%) using this multidrug combination approach in locally advanced gastric cancer.^8^ Considering the similar tumor microenvironment of gastrointestinal tumors, it is rational to combine antiangiogenic drugs with chemoimmunotherapy in mPC.

Penpulimab, an anti-PD1 IgG1 antibody, has been shown to improve efficacy and a low incidence of immune-related adverse events (irAEs).^9^ In cases of mPC, it has demonstrated a partial response (PR) rate of 11.1% (1/9) and no adverse events (AEs) of grade ≥3. Anlotinib, an angiogenesis inhibitor known for its significant cytotoxicity on PC cells, could enhance anti-PC activity when used in conjunction with anti-PD1.^10,11^ GS regimen (gemcitabine, tegafur-gimeracil-oteracil potassium) plus anlotinib revealed a longer OS compared with chemotherapy alone in mPC.^12^ Additionally, comprehensive detection of the tumor immune microenvironment (TIME) might contribute to the identification of predictive biomarkers for mPC patients receiving combination therapy.

Herein, we report the clinical and translational results of a prospective, multicentre, single-arm, phase II trial for the first-line treatment of mPC patients who received PAAG. This clinical study was accompanied by multiomics profiling to identify various distinct biomarkers specific to the treatment.

## Results

### Patients

**Fig. 1:**
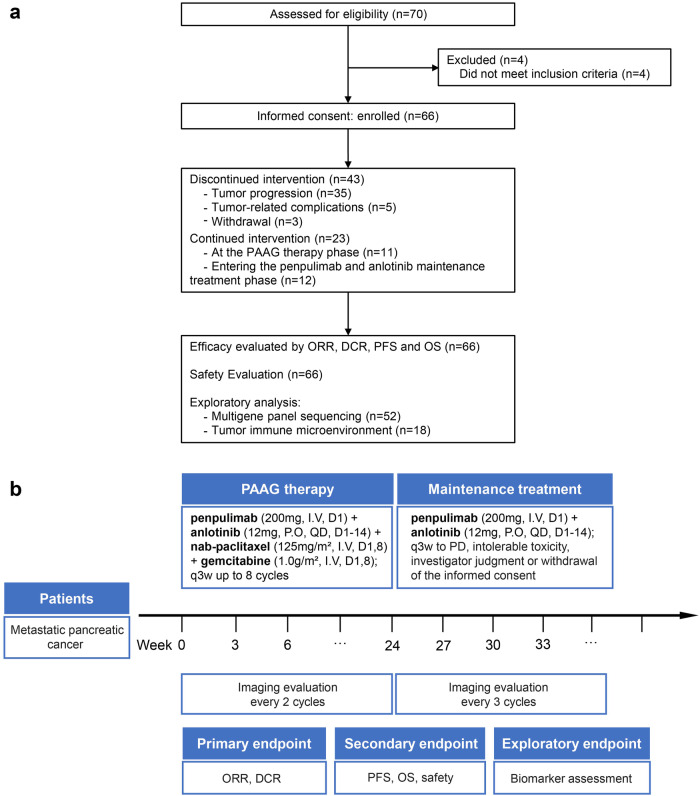
PAAG study flow diagram. **a** Patient dispositions. Seventy participants were screened for eligibility, and 66 were enrolled in the PAAG study. Treatment efficacy was evaluated by objective response rate (ORR), disease control rate (DCR), median progression-free survival (mPFS) and median overall survival (mOS) (full analysis set, *n* = 66). Safety evaluation was performed (*n* = 66). Exploratory analysis was performed by multigene panel sequencing (*n* = 52) and tumor immune microenvironment (*n* = 18). **b** The diagram of the timeline shows the treatment regimen tested with eight cycles of PAAG until progression or intolerable toxicity

Between July 2022 and November 2023, 66 patients with mPC from four hospitals in China were enrolled in the PAAG study (Fig. 1a, b). Each patient was analysed for safety and efficacy. The patients that were included had a median age of 60.0 years, were predominantly male (62%, 41/66), and had a 70% incidence of liver metastases (46/66) (Table 1). The median follow-up period at the analysis’s data cut-off date of February 15, 2024, was 13.3 months. At the time of data cutoff, 66 patients were being monitored, with the study’s treatment ongoing in 23 patients (34.8%). There are 12 patients entering the maintenance treatment phase with penpulimab and anlotinib (Fig. 1a). Among patients who discontinued treatment (*n* = 43), the reasons for discontinuation were progressive disease (PD) (*n* = 35; 81.4%), patient withdrawal (*n* = 3; 7.0%), and tumor-related complications (*n* = 5; 11.6%), including one patient with intestinal obstruction, two patients with obstructive jaundice, and two patients with gastrointestinal haemorrhage.

**Table 1:** Baseline demographics and clinical characteristics

Characteristic	Patients received PAAG (*N* = 66)
Median age, years (range)	60 (32–79)
Sex, *n* (%)	
Male	41 (62.1)
Female	25 (37.9)
ECOG PS score, *n* (%)	
0	14 (21.2)
1	51 (77.3)
2	1 (1.5)
BMI, *n* (%)	
<24	48 (72.7)
≥24	18 (27.3)
Tumor location, *n* (%)	
Head/neck	26 (39.4)
Body/tail	40 (60.6)
Select sites of metastatic disease, *n* (%)	
Liver	46 (69.7)
Extrahepatic	20 (30.3)
Baseline CA199, *n* (%)	
>27	56 (84.8)
≤27	10 (15.2)
Baseline CA125, *n* (%)	
>30	42 (63.6)
≤30	24 (36.4)
Baseline CEA, *n* (%)	
>10	23 (34.8)
≤10	43 (65.2)
Baseline CA724, *n* (%)	
>6.9	25 (37.9)
≤6.9	41 (62.1)
Baseline CA242, *n* (%)	
>10	51 (77.3)
≤10	15 (22.7)
Pathological specimen types, *n* (%)	
Histology	52 (78.8)
Cytology	14 (21.2)

### Efficacy

**Fig. 2:**
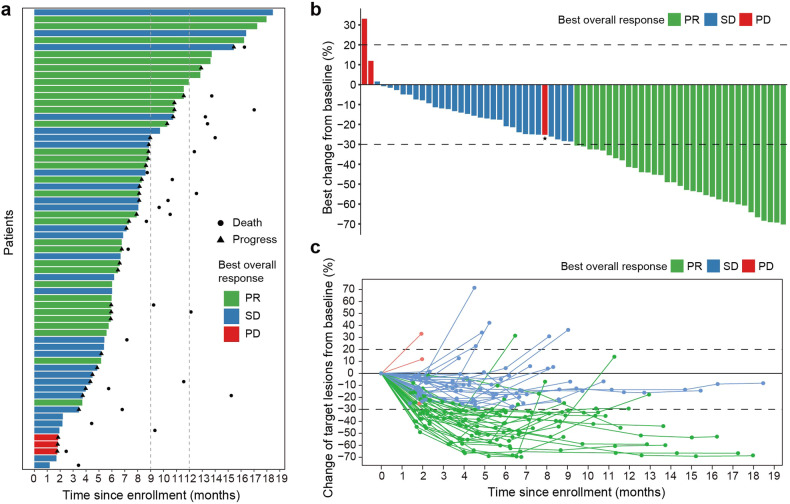
Efficacy of PAAG. **a** A swimmer plot illustrating PFS for the entire cohort (*n* = 66), with colours indicating the best overall response (BOR). Partial response (PR), stable disease (SD), and progressive disease (PD) are shown in green, blue, and red bars, respectively. The length of the bars represents the duration from enrolment to PD. Death and progression events are represented by circles and triangles, respectively. **b** Waterfall plot of the best percent change in target lesion diameter from baseline, coloured according to BOR (*n* = 66). The pentagram represents that the patient showed a decrease in the diameter of the target lesion, but due to the presence of cancerous ascites, the overall evaluation indicated PD. **c** Spider plot of the change in target lesion diameter from baseline (*n* = 66)

**Fig. 3:**
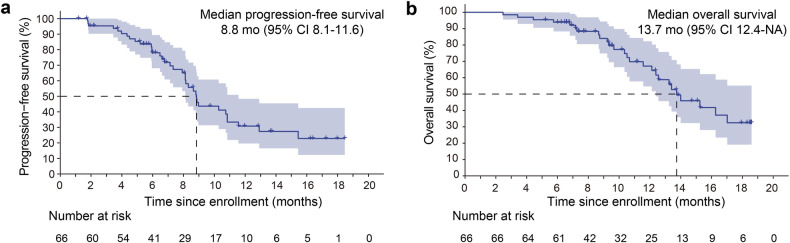
Kaplan–Meier analysis of PFS and OS in PAAG**. a** Kaplan–Meier estimation of PFS for participants who enrolled in the PAAG trial (full analysis set, *n* = 66). **b** Kaplan–Meier estimation of OS for participants who enrolled in the PAAG trial (full analysis set, *n* = 66)

All 66 patients were eligible for response evaluation, and the longest treatment period was nearly 18 months. The best response, indicated by the ORR, was recorded at 50.0% (33/66) (95% CI, 37.4–62.6%), with 33 patients achieving PR. Notably, the disease control rate (DCR) was 95.5% (63/66) (95% CI, 87.3–99.1%) (Fig. 2a–c). In most individuals, reductions from baseline were observed. The mPFS was calculated as 8.8 (95% CI, 8.1–11.6) months (Fig. 3a). For those patients enrolled for more than 1 year, there were 36 individuals, among whom 18 (50%) had a PFS exceeding 8.8 months. The estimated PFS rates at 9 and 12 months were 43.7% (95% CI, 31.4–60.7%) and 30.8% (95% CI, 19.6–48.4%), respectively (Fig. 3a). At the time of cut-off, 26 (39.4%) of 66 patients had died, and the mOS was 13.7 months (95% CI, 12.4 months to not reached) (Fig. 3b). The estimated OS rates at 9 and 12 months were 84.0% (95% CI, 74.9–94.3%) and 67.1% (95% CI, 54.7–82.3%), respectively (Fig. 3b).

### Safety

Overall, all patients experienced AEs. The most common AEs of any grade were anaemia (*n* = 56; 84.8%), leukocytopenia (*n* = 45; 68.2%), fatigue (*n* = 45; 68.2%), and rash (*n* = 44; 66.7%; Supplementary Table 1). Grade 3 or grade 4 treatment-related AEs (TRAEs) were observed in 26 out of 66 patients (39.4%). The most frequently occurring grade 3 or grade 4 TRAEs were neutropenia (*n* = 14; 21.2%) and leukocytopenia (*n* = 14; 21.2%). No grade 5 TRAEs were reported in this trial. The toxicities were mainly associated with chemotherapy. irAEs occurred in 4 patients (6.1%) with cystitis (*n* = 1; 1.5%), pneumonia (*n* = 2; 3.0%) and immunological liver damage (*n* = 1; 1.5%). One patient discontinued immunotherapy due to immunological liver damage of grade 3 and only received chemotherapy and antiangiogenic therapy. Other irAEs were grade 1 and 2 in severity.

### Exploratory analysis of clinical factors

In an exploratory subgroup analysis, we examined clinical prognostic variables. Interestingly, patients with hepatic metastases (Supplementary Fig. 1a) or high levels of serum CA724 ( > 6.9 U/mL) (Supplementary Fig. 1b) had worse PFS. No significant differences in terms of PFS were found for other baseline characteristics (Supplementary Fig. 1c–l).

### Mutational landscape and predictive biomarkers

**Fig. 4:**
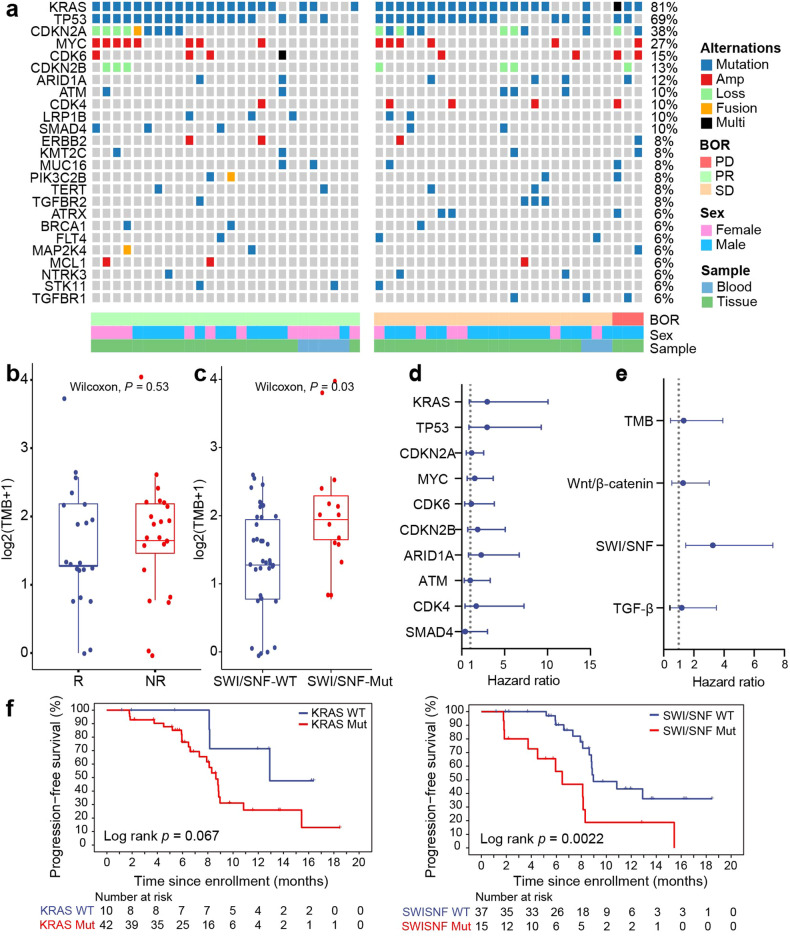
Genomic features of mPC patients (*n* = 52)**. a** Mutational landscape of frequently altered genes. **b** Boxplot of TMB distribution in responder (R) and non-responder (NR) tumors. **c** Boxplot of SWI/SNF wild-type and mutant tumors. **d** HR values with 95% CI for the correlation of gene mutation factors with PFS. **e** HR values with 95% CI for the relationship of pathway features with PFS. **f** Kaplan–Meier curve for PFS in patients harbouring *KRAS* or SWI/SNF complex alterations. Mut, mutant. WT, wild-type. *P* values in (**b**) were compared between R and NR using Wilcoxon rank-sum test. *P* values in (**c**) were compared between SWI/SNF wild-type and mutant using Wilcoxon rank-sum test. All hazard ratios (HR) were computed using the Cox proportional hazards model (**d**, **e**). *P* values in (**f**) were compared between *KRAS* or SWI/SNF complex alterations and non-alterations using log-rank test

Genomic testing was performed in 52 patients, who were divided into the responder (R) and non-responder (NR) groups with a distribution of 26:26 according to their outcomes. *KRAS* was altered most frequently (81%), followed by *TP53* (69%), *CDKN2A* (38%), *MYC* (27%) and *CDK6* (15%) (Fig. 4a). No significant differences in tumor mutation burden (TMB) values (Fig. 4b), variant frequency in single nucleotide variants (SNVs), copy number variants (CNVs) and key signalling pathways, such as the SWI/SNF complex, TGF-β and Wnt-β catenin (Supplementary Fig. 2), were observed between the R and NR groups. Interestingly, we found that patients harbouring *KRAS* mutations (HR 3.0, 95% CI 0.9–10.1) or SWI/SNF (HR 3.3, 95% CI 1.5–7.2, *P* = 0.002) alterations had shorter PFS than wild-type patients (Fig. 4d–f), and higher TMB values were identified in SWI/SNF mutators than in wild-type patients (*P* = 0.030, Fig. 4c).

### Pre-PAAG TIME between R and NR lesions

**Fig. 5:**
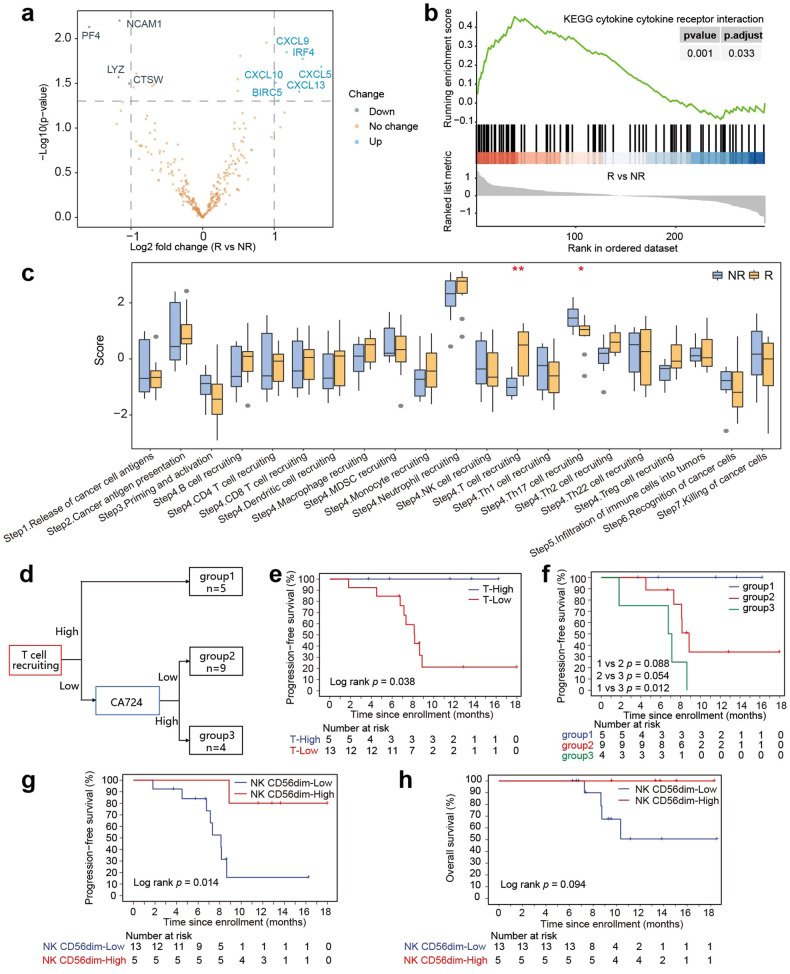
Transcriptomic profiling between responder (R) and non-responder (NR) patients (*n* = 18)**. a** Differential immune-related gene expression in R/NR tumors, as described by volcano plot. **b** GSEA pathway enrichment analysis of differentially expressed genes between R and NR patients. **c** Comparison of the cancer-immunity process between R and NR. **d** Decision tree for PFS evaluated with T cell recruitment score (TCRS) and serum CA724 level. **e** Kaplan–Meier analysis of decision tree-guided stratification using T-cell recruiting scores for PFS in patients. **f** Kaplan–Meier analysis of decision tree-guided stratification using both TCRS and CA724 for PFS in patients. **g**, **h** Kaplan–Meier curves illustrating PFS (**g**) and OS (**h**) based on the NK CD56dim score with a cut-off at the upper quartile. *P* values in (c) were compared between R and NR using Wilcoxon rank-sum test. *P* values in (**e**) were compared between T-cell recruiting scores high and T-cell recruiting scores low using log-rank test. *P* values in (**f**) were compared among three groups according to decision tree using log-rank test. *P* values in (**g**, **h**) were compared between NK CD56dim score high and NK CD56dim score low using log-rank test

Among all enrolled patients, programmed cell death-ligand 1 (PD-L1) immunohistochemical testing was conducted in 46 patients, and 10 positive cases were observed. PD-L1 positive expression was not observed to be significantly associated with response and PFS (Supplementary Fig. 3). Transcriptional profiling of baseline tumor tissue specimens from 18 patients (R:NR = 10:8) was performed to explore the potential mechanisms and efficacy biomarkers of PAAG. Compared with NR patients, 4 genes had significantly downregulated expression, including *CTSW*, *LYZ*, *NCAM1* and *PF4*, and 6 genes had significantly upregulated expression, including *BIRC5*, *CXCL5*, *CXCL9*, *CXCL10*, *CXCL13* and *IRF4* (Fig. 5a). The KEGG pathway analysis revealed that responsive tumors had an enrichment of cytokine‒cytokine receptor interactions (Fig. 5b). In total, TIME cell infiltration, TIME signature scores and immune checkpoint gene expression were similar in both populations (Supplementary Fig. 4a–c).

### T cell recruitment score and serum CA724 level in predictive of clinical efficacy

To analyse the cancer-immunity cycle in R and NR patients, which was a series of stepwise events for an antitumor immune response to lead to cancer cell killing, we mapped out this cycle for individual patients. In responding tumors, the T cell recruitment score (TCRS) was significantly higher, and Th17 cell recruiting was significantly lower (Fig. 5c). A decision tree was constructed based on TCRS and CA724 level to further verify the prediction performance for PFS (Fig. 5d). TCRS used the first quartile value (score ≥0.54) as the cut-off of the high group. Patients with high TCRS revealed a significantly prolonged PFS (*P* = 0.038) (Fig. 5e). On contrast, patients with low TCRS and high CA724 had worst PFS (Fig. 5f). These results suggested that both TCRS and CA724 may be predictive biomarkers for PAAG. Additionally, low NK CD56dim cell scores were found to be associated with a shorter PFS (*P* = 0.014) and tended to be correlated with poorer OS (*P* = 0.094) (Fig. 5g, h).

### TIME signatures between PFS-long and PFS-short groups

**Fig. 6:**
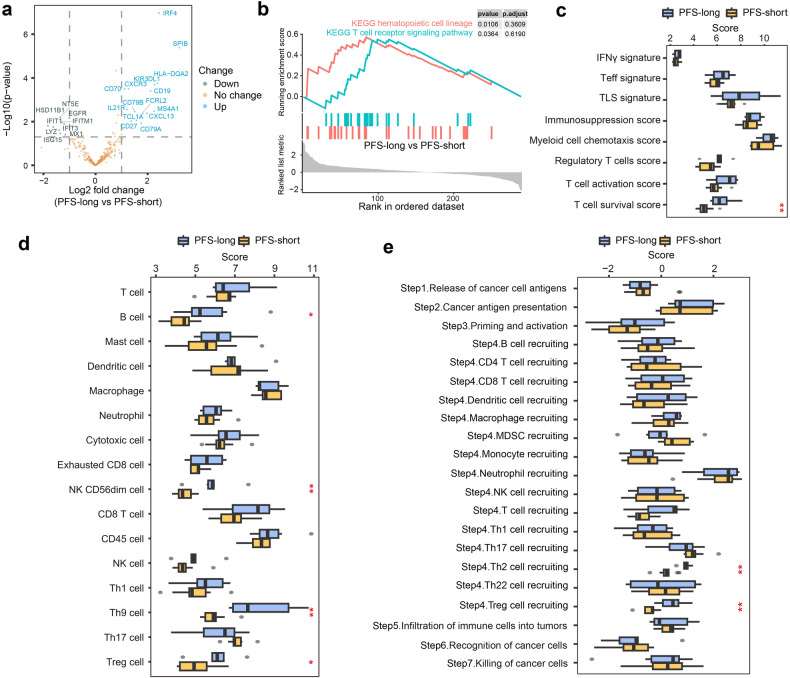
Transcriptomic profiling between PFS-long and PFS-short patients (*n* = 15). **a** Volcano plot describing differential gene expression pattern analysis between PFS-long and PFS-short patients. The log_2_-fold change indicated the mean expression level for each gene. **b** Enriched pathways of cancer hallmarks between the PFS-long and PFS-short groups. **c** The expression differences of immune-related signatures were compared. **d** The expression differences of immune cell scores were compared. **e** The expression differences of cancer-immunity process were compared. Two-sided *P* value was calculated with a significance level set at 0.05. *P* values in sections (**c**–**e**) were compared between PFS-long and PFS-short using Wilcoxon rank-sum test

Patients who were treated with the PAAG regimen for less than 8.8 months without disease progression were excluded. We performed an analysis of the TIME differences between PFS-long and PFS-short patients in 15 patients (6:9) who received the PAAG regimen and were distinguished by mPFS (8.8 m) as the cut-off value. Immune-related mRNA expression profiling, pathway enrichment, and TIME signatures were also analysed in these groups. Fifteen genes were significantly upregulated, and 9 genes were significantly downregulated (Fig. 6a). Both T-cell receptor signalling and hematopoietic cell lineage scores were significantly enriched in PFS-long patients (Fig. 6b). The scores of NK CD56dim cells, B cells, Th9 cells, T-cell survival, Th2 cell recruitment, Treg cells, and Treg cell recruitment were significantly higher in PFS-long group (Fig. 6c–e). The results indicated that these scores appeared to be biomarkers for identifying patients who could benefit from PAAG, especially NK CD56dim cell score, TCRS and CA724 level.

## Discussion

Our first-line PAAG study reaches its primary objective with 50% ORR and 95.5% DCR and displays an acceptable safety profile. To our knowledge, the PAAG study reported a breakthrough response rate and clinical efficacy for first-line treatment of mPC, and our findings provide a novel and encouraging therapeutic strategy for mPC.

In the PAAG study, the PFS reached 8.8 months, and another noteworthy feature is that 50% of the patients in our study experienced PR. In previous chemotherapy studies, the expected mPFS was only 5–6 months, and the rates of PFS exceeding 9 months were only 25% and 29.5% with the use of AG alone (MPACT) or FOLFIRINOX alone.^2,3^ Moreover, in the recently reported CISPD3^4^ (NCT03977272) and PRINCE^5^ (NCT03214250) studies using ICI plus chemotherapy, the mPFS was approximately 5.9 and 6.4 months, respectively. The OS rate at 1 year, was 67.1% in the PAAG study and 57.7% in the PRINCE study. Besides its effectiveness, our triple-combo treatment led to manageable AEs for patients, largely because patients do not need to undergo long cycles of chemotherapy. None of the grade 3 or 4 TRAEs occurred during the maintenance treatment phase, and such a treatment strategy is also one of the innovations of our study.

It is becoming evident that the efficacy of immunomodulatory strategies depends on the existence of a baseline immune response and the activation of preexisting immunity.^13^ Analyses of extensive PC genomic datasets have revealed that only a portion of cases exhibit immunological activity, suggesting that the efficacy of immunotherapy may be related to the immune status.^14,15^ Clinical, genomic and transcriptomic analyses were executed to explore biomarkers via pretreatment specimens of efficacy response and survival for PAAG in our study. Patients with liver metastasis showed significantly worse PFS than extrahepatic metastasis patients, which was consistent with a previous study.^16^ Reportedly, CA724, a serum marker for gastrointestinal tumors, has been shown to predict the effectiveness of adjuvant chemotherapy and chemoimmune combination therapy in gastric cancer in previous studies.^17^ Nevertheless, its predictive value in pancreatic cancer treatment remains unexplored. Importantly, our study identified a significant association between CA724 and PFS, establishing its potential as a peripheral blood biomarker for forecasting the effectiveness of the PAAG regimen and providing a potentially convenient and non-invasive predictor in clinical practice.

Genomic *KRAS* or SWI/SNF aberrations were observed to be associated with poorer PFS in this study. *KRAS* mutations occur most frequently in mPC and lead to high immunosuppression.^18^ Due to the high variant frequency of *KRAS* in mPC, PAAG, which its better efficacy than standard treatment, is still an effective treatment option for *KRAS* mutators. In terms of the effect of SWI/SNF complex mutations on the efficacy of ICIs, there is controversy in current research. One study suggests that it is positive predictive marker for efficacy,^19^ while others argue that it is not relevant.^20,21^ Similar to our study, *KRAS-*SWI/SNF co-mutators with non-small cell lung cancer receiving ICIs have shown shorter survival.^22^ Neither TMB nor PD-L1 expression was observed to be related to the effectiveness of PD1 inhibitor combined with antiangiogenic agent plus chemotherapy in gastric cancer.^23^ Similarly, there were no significant differences in the expression of PD-L1 and other immune checkpoint molecules between R and NR patients (Supplementary Fig. 4c, d). In total, these results indicated that the predictive biomarkers for this triple-combination regimen needed to be explored beyond TMB and PD-L1.

*NCAM1* and *PF4* were downregulated genes in responding tumors with *P* < 0.01. *NCAM1* encodes CD56, and its downregulation is correlated with NK CD56dim, which is responsible for cytolytic activity and target cell killing.^24^ NK cells activity was limited due to the hypoxia caused by changes in vascularization.^25^ The reduction in NK CD56dim cell infiltration was related to abundant H_2_O_2_ in the TIME of digestive cancers. Compared with CD56bright cells, cytotoxic CD56dim cells were more susceptible to H_2_O_2_-induced apoptosis.^26^ PD-1 was overexpressed in NK cells in digestive tumors, and PD-1 antibody could restore NK function in vitro.^27^ In total, the cytotoxic activity of NK CD56dim cells might be preserved by antiangiogenic combined with anti-PD-1 treatment.

A histopathological characteristic of mPC is the surrounding desmoplastic stroma in TIME, induced by activated pancreatic stellate cells stimulated by cancer cells, leading to fibrosis.^28^ Desmoplasia prevents an appropriate vascularization of TIME, restricts chemotherapy exposure, and results in a lack of immune cell infiltration caused by mechanical barriers around the tumor cell.^29,30^ Antiangiogenic agents normalize tumor blood vessel structure and promote T-cell infiltration into the tumor (cell recruitment).^31–33^ ICIs were efficient only when T cells were recruited within the tumors.^34^ Combined CA724 and TCRS enhanced the predictive ability to estimate the efficacy in better selection of mPC patients for antiangiogenic combined chemoimmunotherapy.

Interestingly, patients in the PFS-long group possessed a significantly higher Treg score, while the effect of Treg was augmented by angiogenesis factors that drove immune suppression.^35^ *PF4*, which was downregulated in our responding patients, was verified to suppress angiogenesis by inhibiting endothelial cell proliferation and migration.^36^ This finding suggested that patients with abnormally vascularized tumors appeared to benefit more from antiangiogenic therapy, which could enhance ICIs efficacy by reshaping the suppressive TIME through decreasing the activity of Treg and promoting T-cell recruitment by normalizing the vascular structure.^37^ In conclusion, biomolecular exploration analysis indicated that PAAG appeared to be a more efficient treatment, especially for mPC with a baseline TIME not suitable for ICI monotherapy or ICI plus chemotherapy combined treatment.

This phase II trial has several limitations. First, the follow-up period was relatively short, the OS data were not sufficiently mature made it uncertain whether this regimen confers long-term survival benefits. Second, this study was a single-arm, phase II trial conducted without a control group, which may compromise the robustness of the findings and increase the possibility of comparison errors. An additional chemotherapy control arm will verify the predictive value of detected biomarkers in PAAG. Thus, we are presently undertaking a phase III, randomized, open-label, multicentric clinical trial (NCT06051851) to further analyse the effects, safety, and biomarkers of PAAG as first-line therapy in mPC patients in comparison to those of AG.

In summary, we are the first to report the favourable clinical efficacy of ICIs plus antiangiogenic treatment combined with chemotherapy in first-line mPC. In-depth molecular and immune analysis provides new insights for better selection of late-stage pancreatic cancer patients for the novel regimen, potentially holding broad clinical significance.

## Materials and methods

### Study design and patients

The PAAG study is a prospective, multicentre, single-arm, phase II study that enrolled patients from Nanjing Drum Tower Hospital, the Affiliated Hospital of Jiangsu University, the First Affiliated Hospital of Soochow University, and the Affiliated Jiangning Hospital of Nanjing Medical University between July 2022 and November 2023 (Fig. 1a). Patients meeting the inclusion criteria were 18 years of age or older; had an Eastern Cooperative Oncology Group (ECOG) performance status of 0-1, and demonstrated adequate organ function as determined by laboratory assessments; had histologically or cytologically confirmed mPC; and had treatment-naïve disease. Furthermore, patients exhibited measurable lesions according to RECIST version 1.1 criteria. The study protocol and statistical analysis plan (SAP), detailing the complete eligibility criteria, are provided in Supplementary materials.

This study adhered to the principles outlined in the Declaration of Helsinki and Good Clinical Practice and informed consent was obtained from all patients. Ethical committee approval was obtained at the Ethics Committee of Nanjing Drum Tower Hospital, Affiliated Hospital of Medical School, Nanjing University and each participating site (No.2022-351-02). The study was preregistered with ClinicalTrials.gov (identifier: NCT05493995).

### Procedures

Patients were administered penpulimab (200 mg, I.V., D1) and anlotinib (12 mg, P.O., QD, D1–14), in combination with nab-paclitaxel (125 mg/m^2^, I.V., D1,8) and gemcitabine (1.0 g/m^2^, I.V., D1, 8), following a 21-day treatment cycle (Fig. 1b).

Eligible subjects received PAAG therapy for eight cycles. After 8 cycles, patients with complete response (CR), PR, or stable disease (SD) continued to receive penpulimab and anlotinib maintenance treatment according to the study protocol, and patients with PD are withdrawn from the study. Reasons for treatment termination include intolerable toxicity, PD, investigator judgement, or patient withdrawal of informed consent. The researcher assessed tumor responses using magnetic resonance imaging (MRI) or enhanced computed tomography (CT) every 6 weeks (with a range of 5–7 weeks) in PAAG. Maintenance treatment was evaluated every 9 weeks. Imaging evaluation is based on the RECIST 1.1 criteria. Safety assessments included physical examinations and laboratory evaluations before every cycle of AG and/or each dose of penpulimab. To control TRAEs, dose modifications of AG and anlotinib were permitted. The Common Terminology Criteria for AEs v 5.0, was used to code AEs.

### Outcomes

The primary end points of the study were ORR and DCR. ORR was defined as the proportion of patients with confirmed CR or PR according to RECIST version 1.1, as determined by independent central radiologic review. DCR was defined as the proportion of patients with confirmed CR, PR or SD according to RECIST version 1.1, also assessed by independent central radiologic review. Secondary end points included PFS, which was defined as the interval between the first dose of study medication and the first documented disease progression according to RECIST version 1.1 by independent central radiologic review, or death from any cause, whichever occurred first; OS was another secondary end point, defined as the interval between the date of the study medication’s first dosage and the date of death from any cause. Additionally, safety and tolerability were assessed as secondary end points.

### Biomolecular exploration

Baseline tumour tissue specimens were obtained as required for biomolecular analysis. PD-L1 expression level, mutational identification, Nanostring based RNA sequencing were available in Supplementary Materials.

### Statistical analysis

This was a non-randomized, single-arm clinical trial. The sample size was determined based on the assumption that the ORR, the primary endpoint of this study, using the standard of chemotherapy (AG regimen) as the historical control, was expected to be 23% as reported in a previous study.^2^ The addition of penpulimab and anlotinib would expect to improve ORR to 40% for patients with mPC. With a significance level of 0.05 and power of 0.8, the required sample size is 59. Accounting for an anticipated dropout rate of 10%, the final required sample size is adjusted to 66.

All patients who received at least one cycle of the treatment regimen were included in the efficacy and safety analyses. Point estimates and exact Clopper-Pearson confidence interval (CI) were reported for the ORR. PFS and OS were estimated using the Kaplan‒Meier method based on the log-rank test. Patients who did not achieve a response were not considered in the analysis of response duration. Patients who had not experienced disease progression or death were censored at the time of their last assessment. Cox proportional hazard regression was performed to evaluate the effects of prognostic factors on survival, and the HR and 95% CI were calculated. The differences in clinical and mutational frequencies were calculated by the two-sided *χ*^2^ or Fisher’s exact test when appropriate. A two-sided Wilcoxon rank-sum test was performed to compare the differences between the two populations. *P* < 0.05 was considered significant for all hypothesis tests. All statistical analyses and graphics were managed by computing software R v4.0.2.

### Supplementary information

Revised supplementary Information clean version

Protocol

## Data Availability

Data are available on request sharing by sending requests to the corresponding author Juan Du (dujuanglyy@163.com), which will need the approval of the institutional ethical committees. Clinical data were not publicly available due to involving patient privacy, but can be accessed from the corresponding author, upon request for 3 years; individual de-identified patient data will be shared for clinical study analyses, and data has been submitted to the China National Center for Bioinformation (https://ngdc.cncb.ac.cn/omix, OMIX ID: OMIX006277). The remaining data are available in the Manuscript and Supplemental Materials.
