# Supplementary material for: First-line penpulimab (an anti-PD1 antibody) and anlotinib (an angiogenesis inhibitor) with nab-paclitaxel/gemcitabine (PAAG) in metastatic pancreatic cancer: a prospective, multicentre, biomolecular exploratory, phase II trial
Source: Signal Transduct Target Ther. 2024 Jun 7;9:143. doi: 10.1038/s41392-024-01857-6 (PMC11156675; doi:10.1038/s41392-024-01857-6)
Supplement: Supplementary file 1 — Revised supplementary Information clean version [file 41392_2024_1857_MOESM1_ESM.docx]

Supplementary Materials for

First-line penpulimab (an anti-PD1 antibody) and anlotinib (an angiogenesis inhibitor) with nab-paclitaxel/gemcitabine (PAAG) in metastatic pancreatic cancer: a prospective, multicentre, biomolecular exploratory, phase II trial

Huizi Sha, Fan Tong, Jiayao Ni, Yi Sun, Yahui Zhu, Liang Qi, Xiaoqin Li, Wei Li, Yan Yang, Qing Gu, Xing Zhang, Chan Zhu, Xiaoxuan Wang, Dongsheng Chen, Baorui Liu^*^, Juan Du^*^

Correspondence to: [dujuanglyy@163.com](mailto:dujuanglyy@163.com), baoruiliu@nju.edu.cn

This PDF file includes:

Methods of biomolecular exploration

Figures. S1 to S3

Tables S1 to S4

Determination of PD-L1 expression level

Formalin-fixed paraffin-embedded (FFPE) tumor tissue sections were used for immunohistochemistry (IHC) (22C3 pharmDx assays) measurement of PD-L1 expression. The PD-L1 combined positive score (CPS) was defined as the percentage of PD-L1-positive cells (tumor cells, lymphocytes, macrophages) of the total number of tumor cells. Positive PD-L1 expression was confirmed as a CPS >1.

DNA sequencing and bioinformatics analysis

Genomic DNA was extracted from FFPE tumor samples with over 20% tumor cells using a DNeasy Tissue kit (Qiagen) and from matched blood using a MagMAXTM DNA Multi-Sample Ultra kit (Thermo Fisher). Targeted next-generation sequencing (NGS) based on a 551 cancer-related gene panel (Table S2) was used to identify the genetic alterations in 52 cases. The KAPA Library Preparation kit was used to construct the library, and the Invitrogen Qubit 4.0 was employed to evaluate the library concentration. Sequencing was performed with an average depth of 1500X by the Illumina NovaSeq 6000 system.

A custom pipeline was established, including read alignment, variant calling, and detection of copy number variations (CNVs) and fusions. Adapter trimming was conducted using fastp (v.2.20.0), and cleaned paired-end reads in FASTQ format were aligned to the human reference genome (hg19) with BWA-mem v.0.7.17, among which selected regions were realigned with ABRA2 v2.21. Single nucleotide variants (SNVs) and short insertion and deletion variants (indels) were both called via VarDict v.1.5.7 and InterVar.^1,2^ CNVkit dx1.1 and FACTERA v1.4.4 were utilized to identify CNVs and fusions, respectively.^3,4^

A total of 334 homopolymer repeat loci with adequate coverage on the panel were selected to determine microsatellite instability (MSI) status via Msisensor.^5^ An MSI score of ≥0.15 was classified as MSI high (MSI-H). Tumor mutational burden (TMB) was calculated by summing all single nucleotide variants (SNVs), insertions and deletions in the coding region of targeted genes, excluding alterations that were synonymous, AF < 0.02 or listed as known mutations in COSMIC. TMB high (TMB-H) was defined as the top 25% value.

RNA sequencing based on NanoString panel

Tumor FFPE samples were collected before treatment from 18 patients whose tissues were available. RNA was isolated using a RNeasy FFPE kit (Qiagen), followed by hybridization of 100 ng RNA into the nCounter platform (NanoString Technologies). Transcriptome analysis was performed based on the expression pattern of 289 immune-related genes, including housekeeping genes (Table S3). Raw data were normalized by nSovler 2.6 software, and gene expression values were log2 transformed.

DEseq2 software was applied to estimate the differentially expressed genes with log_2_ |fold change| >1 and *p* value <0.05. KEGG pathway analysis was performed using gene set enrichment analysis (GSEA). This gene profile covered markers of 16 immune types according to the specification of the manufacturer, including B cells, mast cells (MCs), dendritic cells (DCs), macrophages, neutrophils, cytotoxic cells, T cells, exhausted CD8 cells, CD8 T cells, CD45 cells, Th1 cells, Th9 cells, Th17 cells, NK cells, NK-CD56 cells, and Treg cells.^6^ Seven prognostic and ICI response gene signatures were analysed according to a previous study.^7^ Additionally, genes correlated with the cancer-immunity cycle were also stored in 7 gene sets, including release of cancer cell antigens, cancer antigen presentation, T-cell priming and activation, trafficking of T cells to tumors, infiltration of T cells into tumors, recognition of cancer cells by T cells and killing of cancer cells.^8^ The TIME scores were all calculated as the arithmetic mean of the corresponding genes, and the high scores were defined as the top 25% values (Table S4).

**Reference：**

1. Lai, Z. et al. VarDict: a novel and versatile variant caller for next-generation sequencing in cancer research. *Nucleic Acids Res.* **44**: e108 (2016).

2. Li, Q. & Wang, K. InterVar: Clinical Interpretation of Genetic Variants by the 2015 ACMG-AMP Guidelines. *Am. J. Hum. Genet.* **100**: 267-280 (2017).

3. Talevich, E. et al. CNVkit: Genome-Wide Copy Number Detection and Visualization from Targeted DNA Sequencing. *PLoS Comput. Biol.* **12**: e1004873 (2016).

4. Newman, AM. et al. FACTERA: a practical method for the discovery of genomic rearrangements at breakpoint resolution. *Bioinformatics* **30**: 3390-3393 (2014).

5. Niu, B. et al. MSIsensor: microsatellite instability detection using paired tumor-normal sequence data. *Bioinformatics* **30**: 1015-1016 (2014).

6. Newma, AM. et al. Robust enumeration of cell subsets from tissue expression profiles. *Nat. Methods* **12**: 453-457 (2015).

7. Xia, Y. et al. Efficacy and safety of camrelizumab plus apatinib during the perioperative period in resectable hepatocellular carcinoma: a single-arm, open label, phase II clinical trial. *J. Immunother. Cancer* **10**: e004656 (2022).

8. Chen, DS. & Mellman, I. Oncology meets immunology: the cancer-immunity cycle. *Immunity* **39**: 1-10 (2013).

**
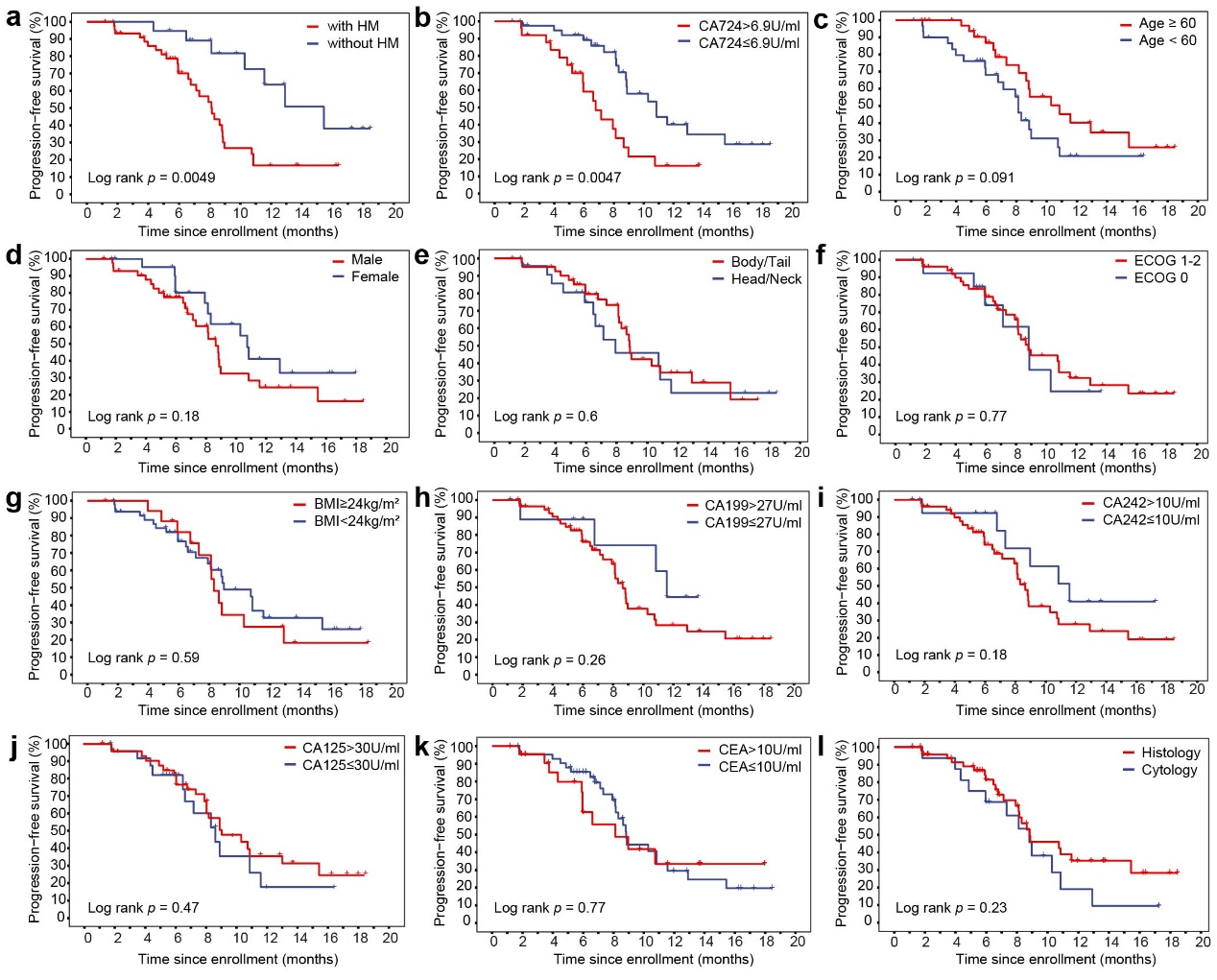
**

**Figure. S1. Evaluation of the clinical characteristics correlated with PFS (n=66). a** Kaplan–Meier curves for PFS with patients stratified according to liver metastases. **b** Kaplan–Meier curves for PFS with patients stratified according to CA724. **c-l** Kaplan–Meier curves for PFS with patients stratified according to baseline characteristics, including age (c), sex (d), tumor location (e), ECOG PS score (f), BMI (g), CA199 (h), CA242 (i), CA125 (j), CEA (k), pathological specimen types (l).


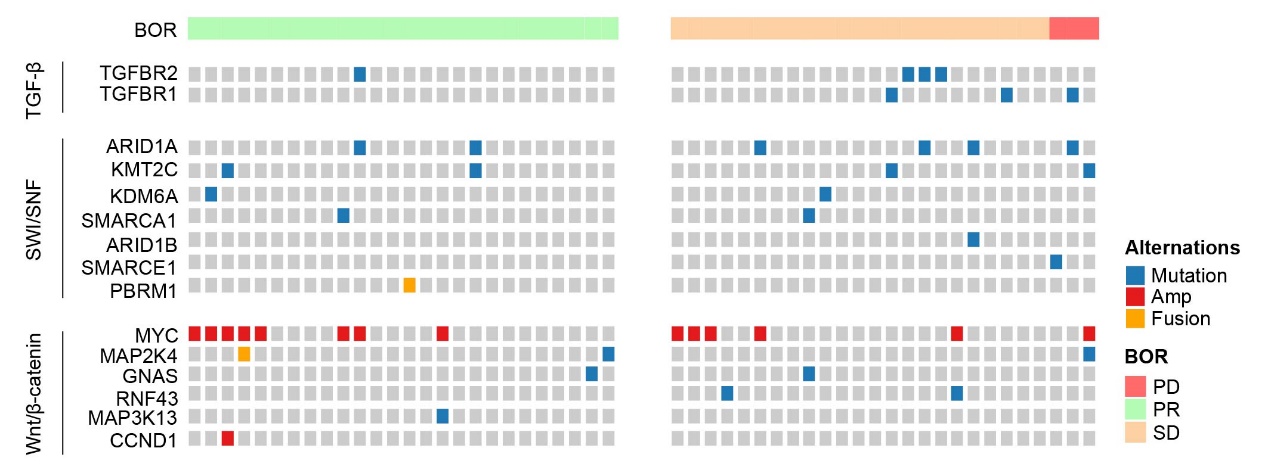


**Figure. S2.** **Mutational distribution of the TGF-β, SWI/SNF complex and Wnt/β-catenin pathways between the R and NR groups (n=52).**


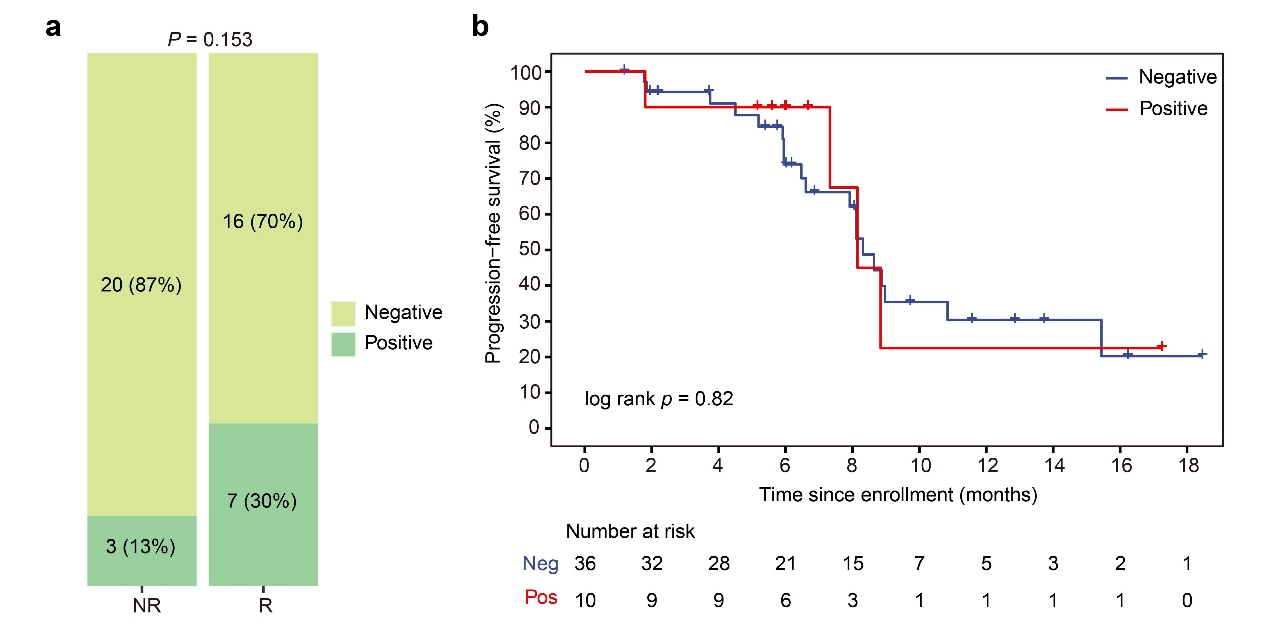


**Figure. S3.** **PD-L1 expression patterns in patients (n=46). a** Positive rate of PD-L1 expression in R/NR patients. **b** Kaplan–Meier curve for PFS with patients stratified based on PD-L1 expression.


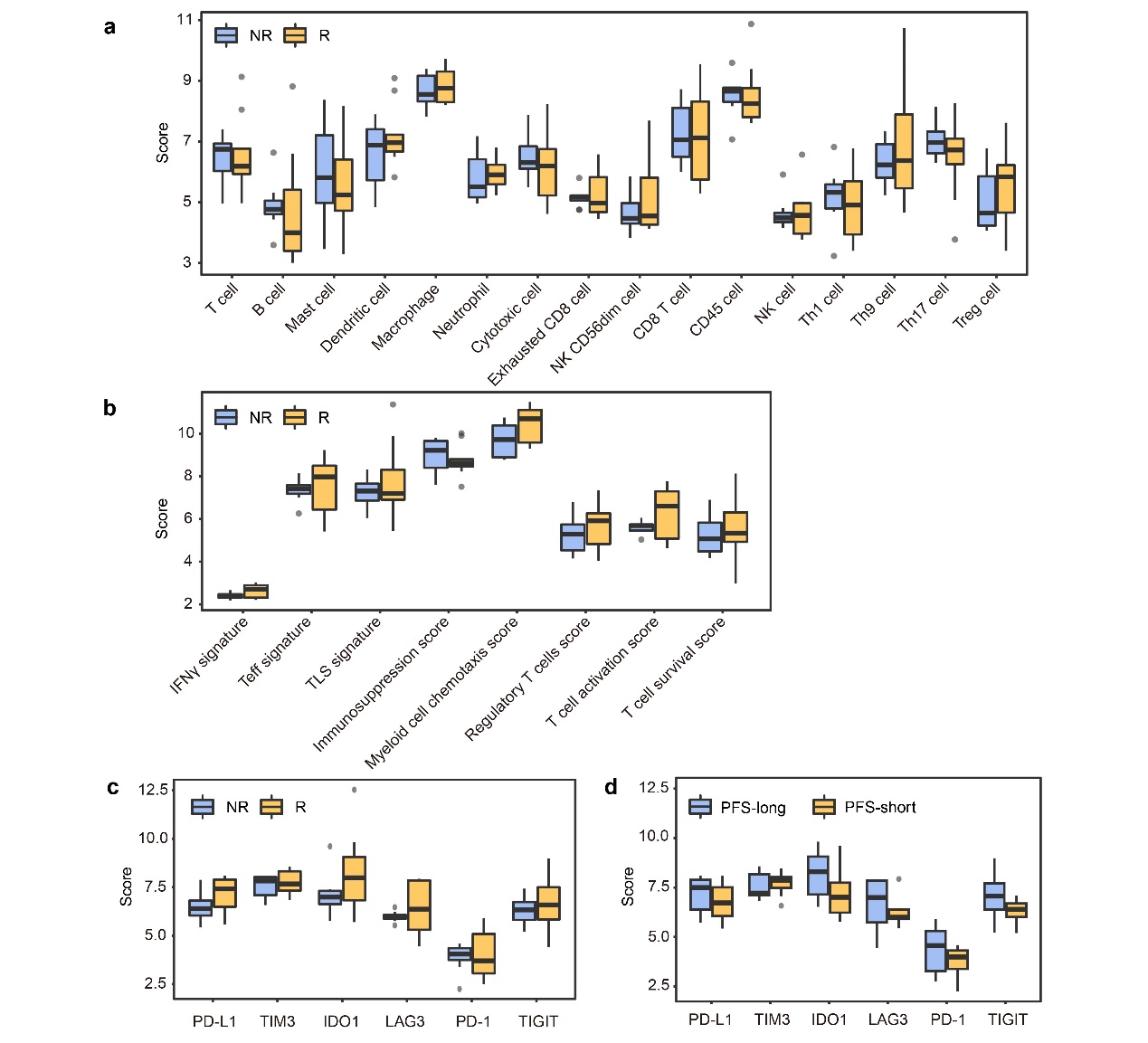


**Figure. S4.** **Exploratory analysis of the differences in TIME. a** The expression differences of immune-related signatures between the R and NR groups were compared. **b** The expression differences of immune cell scores between the R and NR groups were compared. **c** The expression differences of immune checkpoint-related genes between the R and NR groups were compared. **d** The immune checkpoint-related gene expression differences between the PFS-long and PFS-short groups were compared. Two-sided *p* value with significance level set at 0.05. No significant differences were observed between the two groups.

**Table S1.** Summary of adverse events attributed to PAAG

| **Adverse events** | **Any grade n, (%)** | **Grade 3-4 n, (%)** |
| --- | --- | --- |
| Thrombocytopenia | 40 (60.6) | 7 (10.6) |
| Anemic | 56 (84.8) | 2 (3.0) |
| Neutropenia | 42 (63.6) | 14 (21.2) |
| Leukocytopenia | 45 (68.2) | 14 (21.2) |
| Increased AST level | 16 (24.2) | 1 (1.5) |
| Increased ALT level | 21 (31.8) | 0 |
| Vomiting | 18 (27.3) | 0 |
| Nausea | 20 (30.3) | 0 |
| Fatigue | 45 (68.2) | 0 |
| Numbness | 16 (24.2) | 0 |
| Mucositis oral | 14 (21.2) | 1 (1.5) |
| Hyponatremia | 8 (12.1) | 0 |
| Hypoproteinemia | 34 (51.5) | 0 |
| Increased total bilirubin | 8 (12.1) | 0 |
| Rash | 44 (66.7) | 1 (1.5) |
| Insomnia | 21 (31.8) | 0 |
| Poor appetite | 23 (34.8) | 0 |
| Abdominal pain | 37 (56.1) | 0 |
| Hypopotassemia | 7 (10.6) | 0 |
| Fever | 20 (30.3) | 0 |
| Constipation | 15 (22.7) | 0 |
| Epistaxis | 10 (15.2) | 0 |
| Hypothyroidism | 18 (27.3) | 0 |
| Hypertension | 28 (42.4) | 0 |
| Hyperglycemia | 21 (31.8) | 0 |
| Diarrhea | 10 (15.2) | 0 |
| Tinnitus | 5 (7.6) | 0 |
| Proteinuria | 8 (12.1) | 0 |
| ICI-related pneumonitis | 2 (3.0) | 0 |
| ICI-related liver injury | 1 (1.5) | 1 (1.5) |
| ICI-related cystitis | 1 (1.5) | 0 |
| Upper gastrointestinal hemorrhage | 3 (4.5) | 0 |
| Pigmentation | 5 (7.6) | 0 |
| Intestinal obstruction | 3 (4.5) | 0 |
| Hand-foot syndrome | 2 (3.0) | 0 |

**Table S2. A list of 551 cancer-related gene panel**

| **Gene list** | | | | | |
| --- | --- | --- | --- | --- | --- |
| *A2M* | *CDKN2C* | *FGF19* | *JAK2* | *PARP3* | *SMAD2* |
| *ABCB1* | *CEBPA* | *FGF23* | *JAK3* | *PAX5* | *SMAD3* |
| *ABL1* | *CFH* | *FGF3* | *JUN* | *PAX8* | *SMAD4* |
| *ABRAXAS1* | *CFHR1* | *FGF4* | *KDM5A* | *PBRM1* | *SMARCA1* |
| *ACTL6A* | *CHD2* | *FGF6* | *KDM5C* | *PDCD1* | *SMARCA2* |
| *ACTL6B* | *CHD4* | *FGFR1* | *KDM6A* | *PDCD1LG2* | *SMARCA4* |
| *ACVR1B* | *CHEK1* | *FGFR2* | *KDR* | *PDE4D* | *SMARCB1* |
| *ACVR2A* | *CHEK2* | *FGFR3* | *KEAP1* | *PDGFRA* | *SMARCC1* |
| *ADH1B* | *CIC* | *FGFR4* | *KEL* | *PDGFRB* | *SMARCC2* |
| *AKT1* | *CIITA* | *FH* | *KIT* | *PDK1* | *SMARCD1* |
| *AKT2* | *CREBBP* | *FLCN* | *KLF4* | *PGR* | *SMARCE1* |
| *AKT3* | *CRKL* | *FLT1* | *KLHL6* | *PHOX2B* | *SMO* |
| *ALDH2* | *CRLF2* | *FLT3* | *KMT2C* | *PIGR* | *SNCAIP* |
| *ALK* | *CSF1R* | *FLT4* | *KRAS* | *PIK3C2B* | *SOCS1* |
| *ALOX12B* | *CSF3R* | *FOXA1* | *LATS1* | *PIK3C2G* | *SOD2* |
| *AMER1* | *CTCF* | *FOXL2* | *LATS2* | *PIK3C3* | *SOS1* |
| *APC* | *CTLA4* | *FOXO1* | *LIG3* | *PIK3CA* | *SOX10* |
| *APLNR* | *CTNNA1* | *FOXP1* | *LMO1* | *PIK3CB* | *SOX17* |
| *AR* | *CTNNB1* | *FUBP1* | *LRP1B* | *PIK3CG* | *SOX2* |
| *ARAF* | *CUL3* | *FYN* | *LTK* | *PIK3R1* | *SOX9* |
| *ARFRP1* | *CUL4A* | *FZR1* | *LYN* | *PIK3R2* | *SPEN* |
| *ARID1A* | *CXCR4* | *GABRA6* | *MAF* | *PIM1* | *SPOP* |
| *ARID1B* | *CYLD* | *GATA1* | *MAP2K1* | *PLCG2* | *SPTA1* |
| *ARID2* | *CYP17A1* | *GATA2* | *MAP2K2* | *PLK1* | *SRC* |
| *ARID5B* | *CYP19A1* | *GATA3* | *MAP2K4* | *PMS1* | *SRSF2* |
| *ASXL1* | *CYP2C19* | *GATA4* | *MAP3K1* | *PMS2* | *STAG2* |
| *ATG13* | *CYP2C8* | *GATA6* | *MAP3K13* | *POLD1* | *STAT1* |
| *ATG2A* | *CYP2D6* | *GEN1* | *MAPK1* | *POLE* | *STAT2* |
| *ATG7* | *CYP2E1* | *GGH* | *MAPK3* | *POLR2A* | *STAT3* |
| *ATM* | *DAPK1* | *GLI1* | *MAX* | *PPARG* | *STAT4* |
| *ATR* | *DAXX* | *GNA11* | *MCL1* | *PPP2R1A* | *STAT5A* |
| *ATRX* | *DDR1* | *GNA13* | *MDM2* | *PRDM1* | *STAT5B* |
| *AURKA* | *DDR2* | *GNAQ* | *MDM4* | *PREX2* | *STAT6* |
| *AURKB* | *DDX3X* | *GNAS* | *MED12* | *PRKAR1A* | *STK11* |
| *AXIN1* | *DHFR* | *GREM1* | *MEF2B* | *PRKCI* | *SUFU* |
| *AXIN2* | *DICER1* | *GRIN2A* | *MEN1* | *PRKDC* | *SUZ12* |
| *AXL* | *DIS3* | *GRM3* | *MERTK* | *PRKN* | *SYK* |
| *B2M* | *DNMT1* | *GSK3B* | *MET* | *PRSS1* | *TBX3* |
| *BAK1* | *DNMT3A* | *GSTM1* | *MITF* | *PTCH1* | *TCF7L2* |
| *BAP1* | *DOT1L* | *GSTP1* | *MKNK1* | *PTEN* | *TEK* |
| *BARD1* | *DPYD* | *GSTT1* | *MLH1* | *PTK2* | *TENT5C* |
| *BCL10* | *DYNC2H1* | *H3F3A* | *MPL* | *PTPN11* | *TERT* |
| *BCL2* | *EED* | *H3F3B* | *MRE11* | *PTPRB* | *TET1* |
| *BCL2L1* | *EGFR* | *H3F3C* | *MSH2* | *PTPRD* | *TET2* |
| *BCL2L11* | *EML4* | *HDAC1* | *MSH3* | *PTPRO* | *TFG* |
| *BCL2L2* | *EMSY* | *HDAC2* | *MSH6* | *QKI* | *TGFBR1* |
| *BCL6* | *EP300* | *HGF* | *MST1R* | *RAC1* | *TGFBR2* |
| *BCOR* | *EPAS1* | *HIST1H1C* | *MTAP* | *RAC2* | *THADA* |
| *BCORL1* | *EPCAM* | *HIST1H2BD* | *MTHFR* | *RAD17* | *TIPARP* |
| *BIRC3* | *EPHA2* | *HIST1H3A* | *MTOR* | *RAD21* | *TMEM127* |
| *BLM* | *EPHA3* | *HIST1H3B* | *MUC16* | *RAD50* | *TMEM173* |
| *BMPR1A* | *EPHA5* | *HIST1H3C* | *MUTYH* | *RAD51* | *TMPRSS2* |
| *BRAF* | *EPHA7* | *HIST1H3D* | *MYB* | *RAD51B* | *TNFAIP3* |
| *BRCA1* | *EPHB1* | *HIST1H3E* | *MYC* | *RAD51C* | *TNFRSF11A* |
| *BRCA2* | *EPHB4* | *HIST1H3G* | *MYCL* | *RAD51D* | *TNFRSF14* |
| *BRD4* | *ERBB2* | *HIST1H3H* | *MYCN* | *RAD52* | *TNFSF11* |
| *BRD7* | *ERBB3* | *HIST1H3I* | *MYD88* | *RAD54L* | *TOP1* |
| *BRIP1* | *ERBB4* | *HIST1H3J* | *NAT1* | *RAF1* | *TOP2A* |
| *BTG1* | *ERCC1* | *HIST2H3D* | *NBN* | *RARA* | *TP53* |
| *BTG2* | *ERCC2* | *HIST3H3* | *NCOR1* | *RB1* | *TP63* |
| *BTK* | *ERCC3* | *HLA-A* | *NF1* | *RBM10* | *TPMT* |
| *BUB1B* | *ERCC4* | *HLA-B* | *NF2* | *RECQL4* | *TRAF7* |
| *C10orf11* | *ERCC5* | *HLA-C* | *NFE2L1* | *REL* | *TSC1* |
| *CALR* | *ERG* | *HLA-DQB1* | *NFE2L2* | *RELA* | *TSC2* |
| *CARD11* | *ERRFI1* | *HNF1A* | *NFKBIA* | *RET* | *TSHR* |
| *CASP8* | *ESR1* | *HNF1B* | *NKX2-1* | *RHEB* | *TYMS* |
| *CBFB* | *ETV1* | *HOXB13* | *NKX3-1* | *RHOA* | *TYRO3* |
| *CBL* | *ETV4* | *HRAS* | *NOTCH1* | *RICTOR* | *U2AF1* |
| *CBR3* | *EWSR1* | *HSD3B1* | *NOTCH2* | *RNF43* | *UGT1A1* |
| *CCN6* | *EXT1* | *HUWE1* | *NOTCH3* | *ROS1* | *UMPS* |
| *CCND1* | *EXT2* | *ID3* | *NOTCH4* | *RPS6KA3* | *VEGFA* |
| *CCND2* | *EZH2* | *IDH1* | *NPM1* | *RPTOR* | *VEGFB* |
| *CCND3* | *EZR* | *IDH2* | *NQO1* | *RRM1* | *VHL* |
| *CCNE1* | *FANCA* | *IFNGR1* | *NRAS* | *RSPO2* | *VTCN1* |
| *CD274* | *FANCC* | *IFNGR2* | *NRG1* | *RUNX1* | *WAS* |
| *CD70* | *FANCD2* | *IGF1* | *NSD1* | *RXRA* | *WNT10A* |
| *CD74* | *FANCE* | *IGF1R* | *NSD2* | *SBDS* | *WNT10B* |
| *CD79A* | *FANCF* | *IGF2* | *NSD3* | *SDC4* | *WNT7B* |
| *CD79B* | *FANCG* | *IGFN1* | *NT5C2* | *SDHA* | *WRN* |
| *CDA* | *FANCI* | *IKBKE* | *NTHL1* | *SDHAF2* | *WT1* |
| *CDC42* | *FANCL* | *IKZF1* | *NTRK1* | *SDHB* | *XBP1* |
| *CDC73* | *FANCM* | *IL6R* | *NTRK2* | *SDHC* | *XIAP* |
| *CDH1* | *FAS* | *IL6ST* | *NTRK3* | *SDHD* | *XPA* |
| *CDK12* | *FAT1* | *IL7R* | *NUP93* | *SERPINB3* | *XPC* |
| *CDK4* | *FAT2* | *INHBA* | *NUTM1* | *SERPINB4* | *XPO1* |
| *CDK6* | *FAT3* | *INPP4B* | *P2RY8* | *SETD2* | *XRCC1* |
| *CDK8* | *FAT4* | *INSR* | *PAK1* | *SF3B1* | *XRCC2* |
| *CDKN1A* | *FBXW7* | *IRF2* | *PAK3* | *SGK1* | *YAP1* |
| *CDKN1B* | *FGA* | *IRF4* | *PAK5* | *SH2D1A* | *YES1* |
| *CDKN1C* | *FGF10* | *IRS1* | *PALB2* | *SLC34A2* | *ZNF217* |
| *CDKN2A* | *FGF12* | *IRS2* | *PARP1* | *SLCO1B1* | *ZNF703* |
| *CDKN2B* | *FGF14* | *JAK1* | *PARP2* | *SLX4* |  |

**Table S3. A list of 289 immune-related genes in Nanostring RNA sequencing panel**

| **Gene List** | | | | | |
| --- | --- | --- | --- | --- | --- |
| *ABCF1* | *CD44* | *FCGR1A* | *IL21R* | *NFKBIA* | *TIE1* |
| *ADM* | *CD47* | *FCGR2B* | *IL2RA* | *NKG7* | *TIGIT* |
| *ADORA2A* | *CD48* | *FCRL2* | *IL2RB* | *NOS2* | *TLR3* |
| *AKT1* | *CD6* | *FGF13* | *IL2RG* | *NT5E* | *TLR7* |
| *ANGPT2* | *CD68* | *FOXP3* | *IL4* | *OAS1* | *TLR8* |
| *ARG1* | *CD69* | *FPR1* | *IL6* | *OAS2* | *TLR9* |
| *ATM* | *CD70* | *FUT4* | *IL7R* | *OAS3* | *TNF* |
| *AXL* | *CD74* | *G6PD* | *IRF1* | *PDCD1* | *TNFRSF14* |
| *BCL2* | *CD79A* | *GBP1* | *IRF4* | *PDCD1LG2* | *TNFRSF17* |
| *BIRC5* | *CD79B* | *GNLY* | *IRF9* | *PDGFA* | *TNFRSF18* |
| *BLK* | *CD80* | *GUSB* | *ISG15* | *PDGFB* | *TNFRSF1A* |
| *BLM* | *CD84* | *GZMA* | *ITGA1* | *PECAM1* | *TNFRSF1B* |
| *BRCA1* | *CD86* | *GZMB* | *ITGAE* | *PIK3CA* | *TNFRSF4* |
| *BRCA2* | *CD8A* | *GZMH* | *ITGAL* | *PIK3CD* | *TNFRSF9* |
| *BRIP1* | *CD8B* | *GZMK* | *ITGAM* | *PMS2* | *TNFSF10* |
| *BTLA* | *CDKN2A* | *HAVCR2* | *ITGAX* | *PNOC* | *TNFSF13B* |
| *C1QA* | *CEACAM3* | *HDC* | *ITGB2* | *POLR2A* | *TNFSF18* |
| *C1QB* | *CMKLR1* | *HERC6* | *KIR2DL3* | *PRF1* | *TNFSF4* |
| *CCL13* | *CPA3* | *HIF1A* | *KIR3DL1* | *PSMB10* | *TNFSF9* |
| *CCL18* | *CSF1R* | *HLA-DMA* | *KIR3DL2* | *PSMB9* | *TRAT1* |
| *CCL2* | *CSF2* | *HLA-DMB* | *KLRB1* | *PTEN* | *TWIST1* |
| *CCL20* | *CSF2RB* | *HLA-DOA* | *KLRD1* | *PTGER4* | *VCAM1* |
| *CCL21* | *CSF3R* | *HLA-DOB* | *KLRK1* | *PTGS2* | *VEGFA* |
| *CCL22* | *CTAG1B* | *HLA-DPA1* | *LAG3* | *PTPN11* | *VTCN1* |
| *CCL4* | *CTLA4* | *HLA-DQA2* | *LCK* | *PTPRC* | *ZAP70* |
| *CCL5* | *CTSS* | *HLA-DRA* | *LILRB2* | *PVR* | *ZEB1* |
| *CCL7* | *CTSW* | *HSD11B1* | *LY9* | *RAD51* | *CXCL2* |
| *CCND1* | *CX3CL1* | *ICAM1* | *LYZ* | *RB1* | *FCGR3B* |
| *CCR2* | *CX3CR1* | *ICOS* | *MAGEA1* | *RORC* | *GZMM* |
| *CCR4* | *CXCL1* | *ICOSLG* | *MAGEA12* | *RUNX3* | *HLA-DQA1* |
| *CCR5* | *CXCL10* | *IDO1* | *MAGEA4* | *S100A12* | *HLA-DRB1* |
| *CD14* | *CXCL11* | *IFI27* | *MAGEC2* | *S100A8* | *HLA-E* |
| *CD163* | *CXCL12* | *IFI35* | *MELK* | *S100A9* | *OAZ1* |
| *CD19* | *CXCL13* | *IFI6* | *MKI67* | *SDHA* | *PF4* |
| *CD1C* | *CXCL5* | *IFIH1* | *MLANA* | *SELL* | *PRR5* |
| *CD2* | *CXCL8* | *IFIT1* | *MLH1* | *SH2D1A* | *STK11IP* |
| *CD209* | *CXCL9* | *IFIT2* | *MMP9* | *SIGLEC5* | *TBC1D10B* |
| *CD244* | *CXCR2* | *IFIT3* | *MRC1* | *SLAMF7* | *TPSAB1* |
| *CD247* | *CXCR3* | *IFITM1* | *MS4A1* | *SNAI1* | *UBB* |
| *CD27* | *CXCR4* | *IFITM2* | *MS4A2* | *SPIB* |  |
| *CD274* | *CXCR6* | *IFNG* | *MS4A4A* | *STAT1* |  |
| *CD276* | *CYBB* | *IL10* | *MSH2* | *STAT3* |  |
| *CD28* | *DLL4* | *IL10RA* | *MSH6* | *STAT4* |  |
| *CD38* | *EGFR* | *IL12RB2* | *MTOR* | *TAP1* |  |
| *CD3D* | *EIF2AK2* | *IL15* | *MX1* | *TBP* |  |
| *CD3E* | *ENTPD1* | *IL17A* | *MYC* | *TBX21* |  |
| *CD3G* | *EOMES* | *IL18* | *NBN* | *TCL1A* |  |
| *CD4* | *FAS* | *IL1A* | *NCAM1* | *TDO2* |  |
| *CD40* | *FASLG* | *IL1B* | *NCR1* | *TFRC* |  |
| *CD40LG* | *FCAR* | *IL2* | *NECTIN2* | *TGFB1* |  |

**Table S4. Maker genes of TIME-related signatures**

| **Signatures Category** | **Marker Genes** | **Signatures Category** | **Marker Genes** | **Signatures Category** | **Marker Genes** |
| --- | --- | --- | --- | --- | --- |
| T cell | *CD3D* | Immunosuppression | *TGFB1* | trafficking of T cells to tumors | *CCL4* |
| T cell | *CD3E* | Immunosuppression | *TGFB3* | trafficking of T cells to tumors | *CCL5* |
| T cell | *CD3G* | Immunosuppression | *LGALS1* | trafficking of T cells to tumors | *CXCR2* |
| T cell | *CD6* | Myeloid cell chemotaxis | *CCL2* | trafficking of T cells to tumors | *CXCL5* |
| T cell | *SH2D1A* | Regulatory T cells | *FOXP3* | trafficking of T cells to tumors | *CCL2* |
| T cell | *TRAT1* | Regulatory T cells | *TNFRSF18* | trafficking of T cells to tumors | *CCL7* |
| B cell | *BLK* | T cell activation | *CXCL9* | trafficking of T cells to tumors | *CX3CL1* |
| B cell | *CD19* | T cell activation | *CXCL10* | trafficking of T cells to tumors | *CXCL1* |
| B cell | *FCRL2* | T cell activation | *CXCL16* | trafficking of T cells to tumors | *CXCL2* |
| B cell | *MS4A1* | T cell activation | *INFG* | trafficking of T cells to tumors | *CXCL8* |
| B cell | *PNOC* | T cell activation | *IL15* | trafficking of T cells to tumors | *CXCL5* |
| B cell | *SPIB* | T cell survival score | *CD70* | trafficking of T cells to tumors | *CXCR3* |
| B cell | *TCL1A* | T cell survival score | *CD27* | trafficking of T cells to tumors | *CXCL10* |
| B cell | *TNFRSF17* | release of cancer cell antigens | *IL10* | trafficking of T cells to tumors | *CXCL9* |
| Dendritic cell | *CCL13* | release of cancer cell antigens | *TGFB1* | trafficking of T cells to tumors | *CCL4* |
| Dendritic cell | *CD209* | release of cancer cell antigens | *CXCL10* | trafficking of T cells to tumors | *CCL5* |
| Dendritic cell | *HSD11B1* | cancer antigen presentation | *TNF* | trafficking of T cells to tumors | *CXCL11* |
| Macrophage | *CD163* | cancer antigen presentation | *IL1A* | trafficking of T cells to tumors | *CX3CL1* |
| Macrophage | *CD68* | cancer antigen presentation | *IL1B* | trafficking of T cells to tumors | *CXCL9* |
| Macrophage | *CD84* | cancer antigen presentation | *CD40LG* | trafficking of T cells to tumors | *CCL4* |
| Macrophage | *MS4A4A* | cancer antigen presentation | *CD40* | trafficking of T cells to tumors | *CCL5* |
| Mast cell | *CPA3* | cancer antigen presentation | *TLR3* | trafficking of T cells to tumors | *CCL21* |
| Mast cell | *HDC* | cancer antigen presentation | *TLR7* | trafficking of T cells to tumors | *CX3CL1* |
| Mast cell | *MS4A2* | cancer antigen presentation | *TLR8* | trafficking of T cells to tumors | *CXCL13* |
| Neutrophil | *CEACAM3* | cancer antigen presentation | *TLR9* | trafficking of T cells to tumors | *CXCR3* |
| Neutrophil | *CSF3R* | cancer antigen presentation | *TAP1* | trafficking of T cells to tumors | *CXCL10* |
| Neutrophil | *FCAR* | cancer antigen presentation | *IL10* | trafficking of T cells to tumors | *CXCL9* |
| Neutrophil | *FPR1* | cancer antigen presentation | *IL4* | trafficking of T cells to tumors | *CXCL11* |
| Neutrophil | *S100A12* | T cells priming and activation | *CD3D* | trafficking of T cells to tumors | *CCL20* |
| Neutrophil | *SIGLEC5* | T cells priming and activation | *CD3E* | trafficking of T cells to tumors | *CXCL12* |
| Exhausted CD8 cell | *PTGER4* | T cells priming and activation | *CD3G* | trafficking of T cells to tumors | *CXCR4* |
| Exhausted CD9 cell | *LAG3* | T cells priming and activation | *CD247* | trafficking of T cells to tumors | *CCL22* |
| Exhausted CD10 cell | *EOMES* | T cells priming and activation | *CD28* | trafficking of T cells to tumors | *CCL20* |
| Exhausted CD11 cell | *CD244* | T cells priming and activation | *TNFRSF9* | trafficking of T cells to tumors | *CCR4* |
| CD8 T cell | *CD8B* | T cells priming and activation | *TNFSF9* | trafficking of T cells to tumors | *CCL22* |
| CD9 T cell | *CD8A* | T cells priming and activation | *TNFRSF4* | infiltration of T cells into tumors | *STAT1* |
| Cytotoxic cell | *CTSW* | T cells priming and activation | *TNFSF4* | infiltration of T cells into tumors | *ITGB2* |
| Cytotoxic cell | *GNLY* | T cells priming and activation | *CD27* | infiltration of T cells into tumors | *ICAM1* |
| Cytotoxic cell | *GZMA* | T cells priming and activation | *CD70* | infiltration of T cells into tumors | *VEGFA* |
| Cytotoxic cell | *GZMB* | T cells priming and activation | *TNFRSF14* | recognition of cancer cells by T cells | *CD28* |
| Cytotoxic cell | *GZMH* | T cells priming and activation | *CD40* | recognition of cancer cells by T cells | *ICOS* |
| Cytotoxic cell | *KLRB1* | T cells priming and activation | *CD40LG* | recognition of cancer cells by T cells | *ICOSLG* |
| Cytotoxic cell | *KLRD1* | T cells priming and activation | *TNFRSF18* | recognition of cancer cells by T cells | *TNFRSF9* |
| Cytotoxic cell | *KLRK1* | T cells priming and activation | *TNFSF18* | recognition of cancer cells by T cells | *TNFSF9* |
| Cytotoxic cell | *NKG7* | T cells priming and activation | *SLAMF7* | recognition of cancer cells by T cells | *CD27* |
| Cytotoxic cell | *PRF1* | T cells priming and activation | *CD2* | recognition of cancer cells by T cells | *CD70* |
| NK cell | *NCR1* | T cells priming and activation | *CD48* | recognition of cancer cells by T cells | *TNFRSF4* |
| NK CD56dim cell | *KIR3DL1* | T cells priming and activation | *ICOS* | recognition of cancer cells by T cells | *TNFSF4* |
| NK CD57dim cell | *KIR3DL2* | T cells priming and activation | *ICOSLG* | recognition of cancer cells by T cells | *CD40* |
| NK CD58dim cell | *KIR3DL3* | T cells priming and activation | *KLRK1* | recognition of cancer cells by T cells | *CD40LG* |
| NK CD59dim cell | *IL21R* | T cells priming and activation | *CTLA4* | recognition of cancer cells by T cells | *TAP1* |
| CD45 cell | *PTPRC* | T cells priming and activation | *PDCD1* | recognition of cancer cells by T cells | *BIRC5* |
| Treg cell | *FOXP3* | T cells priming and activation | *PDCD1LG2* | recognition of cancer cells by T cells | *MAGEA4* |
| Th1 cell | *TBX21* | T cells priming and activation | *CD274* | recognition of cancer cells by T cells | *PDCD1* |
| Th9 cell | *IRF4* | T cells priming and activation | *TNFRSF14* | recognition of cancer cells by T cells | *PDCD1LG2* |
| Th17 cell | *RORC* | T cells priming and activation | *BTLA* | recognition of cancer cells by T cells | *CD274* |
| IFN-γ signature | *CXCL10* | T cells priming and activation | *HAVCR2* | recognition of cancer cells by T cells | *CTLA4* |
| IFN-γ signature | *CXCL9* | T cells priming and activation | *CD244* | recognition of cancer cells by T cells | *BTLA* |
| IFN-γ signature | *HLA-DRA* | T cells priming and activation | *CD48* | recognition of cancer cells by T cells | *VTCN1* |
| IFN-γ signature | *IDO1* | T cells priming and activation | *TIGIT* | killing of cancer cells | *IFNG* |
| IFN-γ signature | *IFNG* | T cells priming and activation | *LAG3* | killing of cancer cells | *GZMB* |
| IFN-γ signature | *STAT1* | T cells priming and activation | *IL2* | killing of cancer cells | *PRF1* |
| Teff signature | *CD8A* | trafficking of T cells to tumors | *CXCL13* | killing of cancer cells | *PDCD1* |
| Teff signature | *EOMES* | trafficking of T cells to tumors | *CX3CL1* | killing of cancer cells | *VTCN1* |
| Teff signature | *PRF1* | trafficking of T cells to tumors | *CCR5* | killing of cancer cells | *HAVCR2* |
| Teff signature | *INFG* | trafficking of T cells to tumors | *CXCR3* | killing of cancer cells | *BTLA* |
| Teff signature | *CD274* | trafficking of T cells to tumors | *CXCL10* | killing of cancer cells | *LAG3* |
| TLS signature | *CCL19* | trafficking of T cells to tumors | *CXCL9* | killing of cancer cells | *IDO1* |
| TLS signature | *CCL21* | trafficking of T cells to tumors | *CCL20* | killing of cancer cells | *ARG1* |
| TLS signature | *CXCL13* | trafficking of T cells to tumors | *CXCL11* | killing of cancer cells | *NOS2* |
| TLS signature | *CCR7* | trafficking of T cells to tumors | *CX3CL1* | killing of cancer cells | *TGFB1* |
| TLS signature | *CXCR5* | trafficking of T cells to tumors | *CCL4* | killing of cancer cells | *IL10* |
| TLS signature | *SELL* | trafficking of T cells to tumors | *CCL5* | killing of cancer cells | *CXCL12* |
| TLS signature | *LAMP3* | trafficking of T cells to tumors | *CCL21* | killing of cancer cells | *CCL2* |
| Immunosuppression | *CXCL12* | trafficking of T cells to tumors | *CCL2* | killing of cancer cells | *CXCL8* |
