# Supplementary material for: First-line penpulimab (an anti-PD1 antibody) and anlotinib (an angiogenesis inhibitor) with nab-paclitaxel/gemcitabine (PAAG) in metastatic pancreatic cancer: a prospective, multicentre, biomolecular exploratory, phase II trial
Source: Signal Transduct Target Ther. 2024 Jun 7;9:143. doi: 10.1038/s41392-024-01857-6 (PMC11156675; doi:10.1038/s41392-024-01857-6)
Supplement: Supplementary file 2 — Protocol [file 41392_2024_1857_MOESM2_ESM.pdf]

Supplementary Study protocol and Statistical analysis plan

Trial title: The safety and efficacy of **P**enpulimab combined with **A**nlotinib and N**a**b-paclitaxel plus **G**emcitabine (PAAG) as first-line treatment in patients with metastatic pancreatic cancer

Study protocol and statistical analysis plan

Sponsor: Chia Tai Tianqing Pharmaceutical Group Co., LTD

Protocol version number:
Version 1.0, dated 19, February, 2022

CONFIDENTIALITY STATEMENT
The information is provided to you in confidence to enable you to perform the work.
Do not give this document or any copy of it or reveal any proprietary information contained in it to any third party or person without the prior permission of an authorization.

Protocol Signature Page

Investigator's Statement:

I agree to abide by the review comments of the Ethics Committee and start the clinical trial after approval, timely report any changes in the clinical trial activities to the Ethics Committee as well as unexpected problems involving the risks of subjects or other personnel, and re-obtain the approval of ethical review before implementation. Follow-up review and closure review should be performed in accordance with the requirements of the Ethics Committee.

I agree to conduct the clinical trial in strict accordance with the design and specific provisions of this protocol.

I understand that I may interrupt or terminate this clinical trial at any time if it is in the best interest of the subject.

I agree that I will personally conduct or supervise this clinical trial and that all study personnel assisting me in the conduct of this clinical trial at my unit understand their responsibilities in this clinical trial.

I will strictly abide by the current Good Clinical Practice (GCP) and Declaration of Helsinki

________________ ________________ ____________________

Name Signature Date

TABLE OF CONTENTS

Summary 1
1. Background 4
1.1. Disease Background 4
1.2. Research Progress of Immune Checkpoint Inhibitors in PC 4
1.3. Anti-tumor Angiogenesis Therapy in PC 6
2. Study Objectives 7
3. Study Endpoints 7
3.1. Primary Endpoints 8
3.2. Secondary Endpoints 8
3.3. Exploratory Endpoints 8
4. Study Design and Plan 8
4.1. Study Design 8
4.2. Drugs and Treatments Administered 8
5. Patient Population 9
5.1. Inclusion Criteria 9
5.2. Exclusion Criteria 9
5.3. Criteria for Termination of the Study 10
5.4. Withdrawal Criteria 10
6. Determination of Sample Size 10
7. Technical Routes and Processes 10
8. Observation Items and Test Time Points 10
9. Observation of Adverse Events 12
10. Data Safety Monitoring 12
11. General Analysis Considerations 12
11.1. Data Reporting 12
11.2. Data Analysis and Summaries 12
11.3. Data Handling 12
11.3.1. Baseline Characteristics 12
11.3.2. Partial Dates 12
11.3.3. Standard Calculations 13
12. Analysis Populations 13
12.1. Full Analysis Set (FAS) 13
12.2. Safety Set (SS) 13
13. Study Population 13
13.1 Subject Disposition 13
13.2. Protocol Deviations 13
13.3. Demographic and Baseline Characteristics 13
14. Efficacy Analyses 14
14.1. Primary Efficacy Analyses 14
14.1.1. ORR 14
14.1.2. DCR 14
14.2. Secondary Efficacy Analyses 14

14.2.1. OS 14
14.2.2. PFS 14
15. Exploratory Analyses 14
16. Safety Analyses 14
16.1. Adverse Events (AEs) 14
16.2. Clinical Laboratory Evaluations 15
16.3. Vital Signs, physical examinations, and other safety-related examinations 15
16.4. 12-lead Electrocardiograms (ECGs) test 15
17. Ethics of clinical study 15
18. Study Schedule 15
19. References 15

Summary

Study Title	Efficacy and safety of Penpulimab combined with Anlotinib and Nab-paclitaxel plus Gemcitabine (PAAG) as first-line treatment in patients with metastatic pancreatic cancer
Research Purpose	This trial aims to assess the efficacy and safety of penpulimab and anlotinib in combination with nab-paclitaxel plus gemcitabine as first-line therapy in patients with metastatic pancreatic cancer (mPC), and to explore potential biomarkers for predictive therapeutic efficacy.
Study design	A prospective, single-arm, multicenter, phase II clinical trial
Total number of cases	66 patients with metastatic pancreatic cancer<br>The primary endpoint of this single-arm trial was objective response rate (ORR), which was assumed to be 23% based on standard of chemotherapy (AG regimen) as the historical control. It is hypothesized that the incorporation of penpulimab and anlotinib will elevate the ORR to 40% in patients with mPC. With a significance level of 0.05 and a power of 0.8, the required sample size is 59. Accounting for an anticipated dropout rate of 10%, the final required sample size is adjusted to 66. This is determined by a two-sided exact test using PASS 15.0 software.
Criteria for patients enrolled	**Inclusion criteria:**<br>Patients eligible for enrollment in this clinical trial must meet all of the following criteria:<br>1) Age ≥ 18 years; Eastern Cooperative Oncology Group (ECOG) performance status of 0-2; life expectancy ≥3 months;<br>2) Histologically or cytologically confirmed metastatic pancreatic cancer;<br>3) At least one evaluable lesion according to Response Evaluation Criteria in Solid Tumors (RECIST) version 1.1;<br>4) No prior antitumor therapy of any kind;<br>5) Hematological and biochemical parameters must meet the following criteria:<br>➢ White blood cell count (WBC) ≥ 3.0 × 10^9/L;<br>➢ Absolute neutrophil count (ANC) ≥ 1.5 × 10^9/L;<br>➢ Hemoglobin (HB) ≥ 90 g/L;<br>➢ Platelets (PLT) ≥ 75 × 10^9/L;<br>➢ Total bilirubin (TBIL) ≤ 1.5 × upper limit of normal (ULN);<br>➢ Alanine aminotransferase (ALT) and aspartate aminotransferase (AST) ≤ 2.5 × ULN (≤ 5 × ULN for patients with liver metastases);<br>➢ Serum creatinine (Cr) ≤ 1.0 × ULN, or creatinine clearance > 50 ml/min;<br>6) Doppler ultrasound evaluation: left ventricular ejection fraction (LVEF) is ≥ 50% (the lower limit of normal).<br>7) Female should agree to use contraceptive measures (such as intrauterine device [IUD], birth control pill, or condom) during the study and for 6 months after the end of the study. Serum or urine pregnancy test should be negative within 7 days before the study, and patients must be non-lactating. Male should agree to use contraceptive measures during the study and for 6 months after the end of the study period.<br>8) Patients voluntarily participate in this study, sign the informed consent form,

	and demonstrate good compliance with study protocols.<br>**Exclusion criteria:**<br>Patients will be excluded if they meet any of the following criteria:<br>1) Previously treated with systemic anti-tumor therapy, such as chemotherapy, radiotherapy, or other anti-tumor therapies;<br>2) Participated in other drug clinical trial within the previous 4 weeks;<br>3) Patients with surgical opportunity or potential surgical treatment according to the investigator's judgment;<br>4) Patients with moderate ascites requiring drainage (except those with small amounts of ascites on imaging but without symptoms);<br>5) Known presence of symptomatic central nervous system metastases and/or carcinomatous meningitis;<br>6) History of other primary malignancies, except for the following:<br>a. Malignancies in complete remission for at least 2 years before enrollment and without additional treatment during the study;<br>b. Adequately treated nonmelanoma skin cancer or malignant freckle like nevus without evidence of disease recurrence;<br>c. Adequately treated carcinoma in situ without evidence of disease recurrence;<br>7) The patient has autoimmune disease or immunodeficiency and is treated with immunosuppressive drugs;<br>8) The patient has a bleeding tendency;<br>9) Pregnant and lactating women;<br>10) Substance abuse, clinical, psychological or social factors that may interfere with informed consent or conduct of the study.<br>12) Patients with possible allergies to PD-1 monoclonal antibody, anlotinib, albumin-bound paclitaxel, or gemcitabine;<br>**Withdrawal Criteria:**<br>1) During the course of the study, the subject uses other anti-tumor therapies which could affect the judgment of efficacy (including chemotherapy, targeted therapy, or biological agents) ;<br>2) Subjects who experience serious adverse events (AEs) that render them unsuitable for continued participation in the study as determined by the investigator, or in cases of unintended pregnancy;<br>3) Subjects are unwilling to continue in the clinical trial and insist on withdrawing;<br>4) The investigator deems it necessary to stop the subject's involvement in the study.
Study design	Albumin-bound paclitaxel 125mg/m^2 I.V. D1,8<br>Gemcitabine 1.0g/m^2 I.V. D1,8<br>Penpulimab 200mg IV D1<br>Anlotinib 12mg P.O. QD D1-14<br>1 cycle every 21 days<br>The initial efficacy assessment was performed 2 cycles (6 weeks) after dosing. Subsequent efficacy assessments will be conducted every 2 cycles for the

	first 8 cycles of treatment. If treatment extends beyond 8 cycles (24 weeks), efficacy assessments are performed every 3 cycles (9 weeks). Subjects with disease control (complete response [CR], partial response [PR], or stable disease [SD]) and tolerable AEs will continue to receive treatment until efficacy is assessed as disease progression (PD), intolerable AEs occur, or the investigator deems the subject unsuitable for continued treatment.
Effectiveness evaluation indicators	Primary efficacy indicators:<br>1) objective response rate (ORR)<br>2) disease control rate (DCR)<br>Secondary efficacy indicators:<br>1) median overall survival (mOS)<br>2) median progression-free survival (mPFS)<br>3) potential biological indicators for predicting efficacy (tumor tissue NGS test, mRNA, various immune cytokines in peripheral blood, etc.)<br>Safety evaluation indexes<br>Monitoring will include assessment for myelosuppression, nausea, vomiting, liver damage, skin reactions (e.g., rash), pneumonitis, hypertension, proteinuria, fatigue, dyspareunia and other clinical AEs, graded according to the National Cancer Institute (NCI) standard.
Statistical analysis plan	Effectiveness analysis: ORR will be determined by calculating the proportion of subjects in the Full Analysis Set (FAS) with an optimal overall remission of CR or PR. DCR will be determined by calculating the proportion of subjects in the FAS with a best overall remission of CR, PR or SD. For the efficacy indicators OS, PFS, the Kaplan-Meier method will be used to estimate their median time and list the median events and their two-sided 95% confidence intervals.<br>Safety analysis: descriptive statistical analysis will be used to list the AEs observed during the trial. Laboratory test results will be described as normal pre-trial but abnormal post-treatment. This comparison will help in identifying biomarker associated with therapeutic effect.
Evaluation criterions	Safety evaluation: NCI-CTC AE 5.0 will be used to evaluate the AEs of investigational products.<br>Efficacy evaluation: Tumor response will be determined according to RECIST 1.1.
Principal Investigator	Prof. Juan Du
Clinical institution	Nanjing Drum Tower Hospital, Affiliated Hospital of Medical School, Nanjing University<br>the Affiliated Hospital of Jiangsu University,<br>the First Affiliated Hospital of Soochow University,<br>the Affiliated Jiangning Hospital of Nanjing Medical University
Version No.	1.0
Research progress	3 years

1. Background

1.1. Disease Background

Pancreatic cancer (PC) is one of the common malignant tumors of the digestive system, and its rates of morbidity and mortality rates are steadily rising globally. PC is characterized by strong metastasis and invasiveness, which make it difficult to detect in its early stages. Additionally, 85% of the patients are unable to undergo radical surgical treatment at the time of diagnosis, and the prognosis is very poor due to a high rate of metastasis and recurrence after resection. By 2030, PC is predicted to rank second globally in terms of cancer-related deaths, and currently rank the 4th leading cause of malignant tumor death in the United States and the 9th in China [1,2,3].

Systemic chemotherapy is still the cornerstone of advanced PC treatment. Since Gemcitabine (GEM) was launched in 1996 and became the first-line chemotherapy for advanced PC, its status as the only "gold standard" has been unshakeable, but the objective efficiency of gemcitabine as a single agent in advanced PC is only 7% [4]. Combination chemotherapy is often the standard of care for patients with metastatic PC (mPC) who have no possibility of surgery. For first-line treatment in cases of advanced PC, there are now two globally recognized regimens: AG (albumin-bound paclitaxel + gemcitabine) two-drug regimen and FOLFIRINOX (oxaliplatin + irinotecan + calcium folinic acid + 5-FU) four-drug regimen, and both of them have an objective effective rate of about 20%, with an overall survival period is not more than one year[5,6]. Among them, the FOLFIRINOX regimen is more toxic and prone to side effects, and it also demands a higher physical condition of the patient. As a result, people's expectations regarding the effectiveness of advanced PC treatment have not yet been fulfilled. On the basis of the existing standard chemotherapy to further improve the effectiveness of the treatment of late PC, prolong the survival of patients, is an urgent need to explore the direction of research. Additionally, during first-line treatment, patients with advanced PC are advised by the NCCN guideline to actively participate in clinical research [7].

1.2. Research Progress of Immune Checkpoint Inhibitors in PC

In the process of tumor formation and progression, tumor cells have many genetic and epigenetic alterations compared to normal cells, and theoretically have sufficient antigens to be recognized by the body’s immune system, thereby triggering immune response to inhibit tumor growth. However, in practice tumors evade the immune system by suppressing the immune response against them through a number of pathways. There are two main mechanisms for the immune escape flight of the tumor: induction immune tolerance and destroying immune-active cells [8].

In the immune cycle of a tumor, many factors need to work together, including stimulating and suppressing signals. Stimulatory signals enhance immune action, while inhibitory signals play a role in checking and controlling immune activity, attenuating immune activity and tissue autoimmunity function. Typical immune-checking proteins such as CTLA-4 inhibit signals during T-cell growth and proliferation, and Figure 1 shows various stimulatory and inhibitory signals in tumor immune processes, as well as anti-tumor immunotherapy strategies [9].

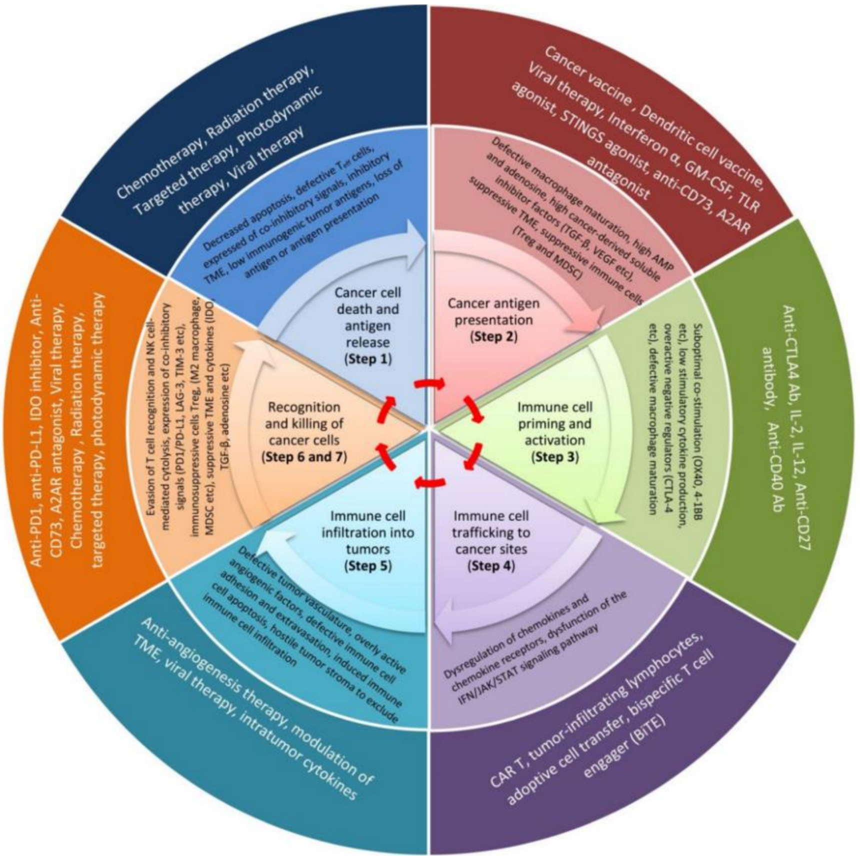

Figure 1 Diverse stimulatory and inhibitory signals in anti-tumor immunotherapy strategies and tumor immune processes

In recent years, tumor immune checkpoint therapy has gradually become a hotspot for research, and has continuously achieved huge breakthroughs. Unlike cytotoxic drugs or monoclonal antibodies targeting tumor driver genes or small molecule tyrosine kinase inhibitors, tumor immune checkpoint therapy does not directly affect tumor cells, but improves the function of T cells by blocking the T cell proliferation and activation inhibiting signals, relieving the immune system's tolerance to tumor cells and improving the effective identification and destruction of T-cells against tumor cells. Tuberculosis immunotherapy target points with significant clinical efficacy have been shown to include cytotoxic T lymphocyte-associated antigen-4 (CTLA-4), and programmed cell death receptor 1/ligand 1 (PD-1/PD-L1). PD-1 is primarily expressed in activating T cells and has two ligands, PD-L1 and PD-L2, of which PD-L1 is the main ligand, and is visible in activated T cells, antigen-presenting cells, and tumor cells. PD-L1 is elevated in a variety of solid tumors, such as lung, liver, gastric, esophageal, uroepithelial, kidney, and other cancers, which is highly correlated with a worse prognosis and shorter survival [10]. The combination of PD-1/PD-L1 plays an important role in regulating T cell activation and maintaining peripheral immune resistance, and if the drug blocks the interaction between PD-1 and PD-L1, it can restore T cell activity and the ability to kill cancer cells.

Numerous monoclonal antibodies that target PD-1 or PD-L1 are in clinical development, and numerous anti-PD-1 and anti-PD-L1 ones have been approved and put on the market both domestically and internationally. In order to treat tumors, anti-PD-1 monoclonal antibodies can specifically bind to PD-1 molecules on the surface of lymphocytes and block the immunosuppressive pathway of PD-1/PD-Ls. This eliminates the tumor cells' ability to evade the immune system and restores the immune system's capacity to identify and destroy tumors. It is generally accepted that anti-PD-1 monoclonal antibodies can block the PD-1, PD-L1, and PD-L2 pathways simultaneously, and therefore may be more effective; in contrast, anti-PD-L1 monoclonal antibodies target the tumor cells and do not bind with

lymphocytes, which may have fewer side effects. Anti-PD-1 monoclonal antibodies are currently approved for the second-line treatment for head and neck tumors, malignant lymphoma, oesophageal cancer, gastric cancer, advanced non-small cell lung cancer, small cell lung cancer, as well as microsatellite unstable tumors and TMB-H solid tumors [11].

A gemcitabine/albumin-bound paclitaxel ± PD-1 monoclonal antibody Nivolumab ± CD40 agonist monoclonal antibody in advanced PC was reported at the 2021 ASCO meeting. A total of 108 patients were enrolled in the study, and the immune-combination chemotherapy had an objective response rate (ORR) of 50%, with an overall survival (OS) rate of 57% at one year, and a median OS (mOS) of 16.7 months [12]. The samples are also being analyzed for multi-omics immunological and tumor biomarkers to reveal possible mechanisms and to identify population subgroups that might profit from combination treatment. A recent study conducted at Nanjing Drum Tower Hospital for the second-line treatment of PC liver metastases after progression on first-line AG chemotherapy with anti-PD-1 monoclonal antibody (sindilizumab) + anlotinib + S-1 (tegafur-gimeracil-oteracil potassium) chemotherapy achieved an ORR of 10.5%, a disease control rate (DCR) of 52.6%, and a mOS of 7.2 months. And this study has been accepted for the AACR meeting in 2022. The efficacy of immune checkpoint inhibitors in PC has dawned, and several studies of PD-1 monoclonal antibody in combination with radiotherapy and other treatments for advanced PC are currently underway [13].

Penpulimab, a PD-1 monoclonal antibody co-developed by Kangfang Bio/ Chia Tai Tianqing Pharmaceutical Group Co., LTD, was formally approved for marketing by the National Medicines and Products Administration (NMPA) on 5 August 2021 for the treatment of relapsed or refractory classical Hodgkin's lymphoma that has been treated with at least second-line systemic chemotherapy [14]. The IgG1 subtype has the advantage of being the most prevalent and structurally stable in human serum, which has implications for both structure and function. Unlike other previously marketed PD-1 monoclonal antibodies, penpulimab is an IgG1 subtype PD-1 monoclonal antibody. It consists of two heavy chains and two light chains of the IgG1 subtype, which are covalently linked to each other by disulfide bonds. The Fc segment of the antibody was designed to eliminate the cytotoxic effects mediated by the Fc receptor and complement, which can cause less damage to target cells and immune cells, increase the therapeutic efficacy and reduce the related adverse reactions. The Fab segment of the antibody was optimized to dissociate more slowly. The preliminary follow-up results showed that ORR 11.1% and DCR 33.3% in the subgroup of PC (N=9) in the phase I/II trail of AK105-204-penpulimab of second-line and above treatment of solid tumors [15]. Based on these research advances, this study proposes to use this drug combination in first-line treatment studies for advanced PC.

1.3. Anti-tumor Angiogenesis Therapy in PC

Although chemotherapy and other anti proliferative drugs can kill tumor cells, due to the support of surrounding blood vessels, residual tumor cells can still obtain blood supply and continue to grow. At the same time, the abnormal tumor vessels reduce drug delivery to the interior of the tumor tissue, which ultimately limits the efficacy of antiproliferative treatments. Therefore, anti-cancer drug treatment strategies not only target the tumor cells, but also target tumor microenvironment, especially tumor angiogenesis, to combat the tumor in an all-round way [16]. Contrary to chemotherapy drugs that only act on tumor cell proliferation, anti-angiogenic drugs exert multiple effects on tumor blood vessels by specifically binding to VEGF and preventing its interaction with receptors. These effects include degrading the tumor's existing blood vessels, which cuts off the oxygen and other nutrients needed for the tumor cells to proliferate; normalizing the tumor's remaining blood vessels and lowering the inter-tumor pressure, to improve the delivery of chemotherapy drugs to the tumor tissue. Additionally, by blocking tumor neo-angiogenesis, this medication increases the effectiveness of chemotherapy, and inhibits tumor neovascularization, thus continuously

inhibiting the growth and metastasis of tumor cells, can maximize the control and killing of tumors.

Anti-angiogenic drugs are currently very effective in the treatment of solid tumors, but their efficacy in the treatment of PC is not satisfactory. Of all the anti-angiogenic drugs, only erlotinib has successfully completed a phase III clinical trial in the treatment of PC. In this phase III trial, which included 569 patients with locally advanced or distantly mPC, demonstrated gemcitabine in combination with erlotinib significantly improved OS and progression-free survival (PFS) of the patients. In contrast to single-agent gemcitabine, the patients' 1-year survival rate was significantly higher (23%) and their median overall survival time was only 2 weeks longer (6.24 months vs. 5.91 months) vs. 17%, P = 0.038) [17]. As a result, the FDA approved the use of gemcitabine in combination with erlotinib for the treatment of unresectable locally advanced or mPC.

In the CALGB80303 study for advanced PC, gemcitabine combined with bevacizumab increased median PFS (mPFS) by 0.9 months extension compared to gemcitabine alone (3.8 months vs. 2.9 months) [18]. One study demonstrated that gemcitabine combined with erlotinib plus bevacizumab could significantly prolong PFS in patients with advanced PC compared to gemcitabine and erlotinib alone [19]. Another study found that gemcitabine in combination with capecitabine, bevacizumab, and erlotinib was safe and effective in treatment of advanced PC with manageable toxicity [20].

Recent study suggest that the combination of immune checkpoint inhibitors and anti-angiogenic agents may be a promising therapeutic strategy to overcome immune checkpoint inhibitor resistance [21]. On the one hand, by inhibiting the direct anti-cancer effect of tumor growth and transmission, anti-vascular generating drugs can reprogram the tumor environment from the immunosuppressive micro-environment to the immuno-permissive micro environment. On the other hand, immune checkpoint inhibitors activate immunity and promote anti angiogenic effects by downregulating the expression of vascular endothelial growth factor and alleviating hypoxia..

Anlotinib is a novel multi-target receptor tyrosine kinase inhibitor that acts on VEGFR1, VEGFR2, VEGFR3, c-Kit, PDGFRβ, Met, FGFR1/2/3 [22]. Anlotinib has been approved for the treatment of lung cancer and soft tissue sarcoma, showing significant efficacy in other tumors as well, and research on its application in PC is gradually progressing [23,24]. Given the aforementioned research progress, this study aims to investigate the combined use of the anlotinib for first-line treatment of mPC.

In recent years, significant progress has been made in the treatment of PC, owing to advancements in tumor immunology and molecular biology technology. Based on the results of previous studies, this research aims to explore the efficacy of combining albumin-bound paclitaxel + gemcitabine chemotherapy with PD-1 monoclonal antibody and anti-angiogenic therapy in the first-line treatment of patients with mPC. Through the detection of patients' clinical performance, pathological characteristics and related indicators such as molecules, genotypes, explore the screening of the potential population that can benefit from the treatment, and provide evidence for the future individualized precision treatment.

2. Study Objectives

This trial aims to assess the efficacy and safety of penpulimab and anlotinib in combination with nab-paclitaxel plus gemcitabine as first-line therapy in patients with mPC, and to explore potential biomarkers for predictive therapeutic efficacy.

3. Study Endpoints

3.1. Primary Endpoints

objective response rate (ORR)

disease control rate (DCR)

3.2. Secondary Endpoints

median progression-free survival (mPFS)

median overall survival (mOS)

patient's safety and tolerability

3.3. Exploratory Endpoints

Correlation between biomarkers and therapeutic response to therapy (including tumor tissue NGS assay, mRNA, various immune cytokines in peripheral blood, etc.)

4. Study Design and Plan

4.1. Study Design

This is a prospective, single-arm, multicentre, phase II clinical study designed to evaluate the clinical efficacy and safety of PAAG as first-line treatment in patients with mPC.

Treatment-naïve patients who pathologically or cytologically confirmed mPC with imaging indications of measurable lesions will be enrolled in this study after signing an informed consent form (ICF).

4.2. Drugs and Treatments Administered

Drug Delivery Flow Chart

Albumin-bound paclitaxel 125mg/m2 I.V. D1,8

Gemcitabine 1.0g/m2 I.V. D1,8

Penpulimab 200mg IV D1

Anlotinib 12mg P.O. QD D1-14

1 cycle every 21 days

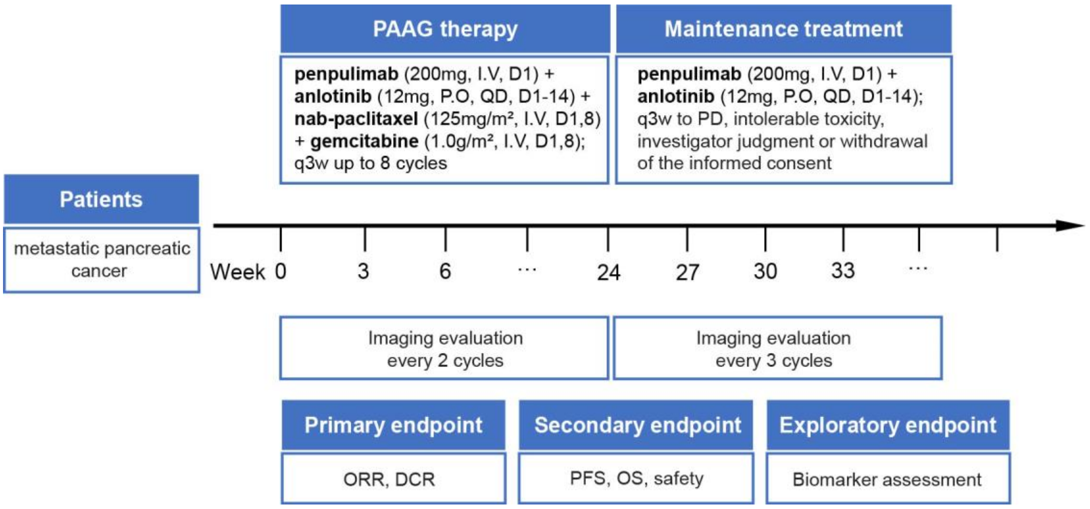

The initial efficacy assessment will be performed 2 cycles (6 weeks) after dosing. Subsequent efficacy assessments will be conducted every 2 cycles for the first 8 cycles of treatment. If treatment extends beyond 8 cycles (24 weeks), efficacy assessments are performed every 3 cycles (9 weeks). Subjects with disease control (complete response [CR], partial response [PR], or stable disease [SD]) and tolerable adverse events (AEs) will continue to receive treatment until efficacy is assessed as disease progression (PD), intolerable AEs occur, or the investigator deems the subject

unsuitable for continued treatment.

5. Patient Population

5.1. Inclusion Criteria

a. Age ≥ 18 years, Eastern Cooperative Oncology Group (ECOG) performance status of 0-2, life expectancy ≥ 3 months.

b. Patients with histologically or cytologically confirmed mPC.

c. Have at least one measurable lesion according to Response Evaluation Criteria (RECIST) version 1.1 criteria;

d. No prior antitumor therapy of any kind.

e. Hematological parameters must meet the following criteria:

e1. White blood cell count (WBC) ≥ 3.0 × 10^9/L;

e2. Absolute neutrophil count (ANC) ≥ 1.5 × 10^9/L

e3. Hemoglobin (HB) ≥ 90 g/L;

e4. Platelet (PLT) ≥ 75 × 10^9/L;

f. Biochemical data shall meet the following criteria:

f1. Total bilirubin (TBIL) ≤ 1.5 × upper limit of normal (ULN)

f2. Alanine aminotransferase (ALT) and aspartate aminotransferase (AST) ≤ 2.5 × ULN, or if accompanied by hepatic metastases, ALT and AST ≤ 5 × ULN;

f3. Serum creatinine (Cr) ≤1.0 × ULN or creatinine clearance (CCr) ≥ 50 ml/min;

g. Doppler ultrasound assessment: left ventricular ejection fraction (LVEF) ≥ 50% (low limit of normal);

h. Female should agree to use contraceptive measures (such as intrauterine device [IUD], birth control pill, or condom) during the study and within for 6 months after the end of the study. Serum or urine pregnancy test should be negative within 7 days before the study, and participants must be non-lactating. Male should agree to use contraceptive measures during the study and within for 6 months after the end of the study period.

i. Patients voluntarily participate in the study, sign the informed consent form, and have good compliance with study protocols and co-operation with follow-up visits.

5.2. Exclusion Criteria

a. Previously treated with systemic anti-tumor therapy, such as chemotherapy, radiotherapy or other anti-tumor therapies

b. Participated in a clinical trial of another drug within the previous 4 weeks;

c. Patients who, in the judgement of the investigator, have access to surgery or are potentially amenable to surgical treatment;

d. Patients with moderate ascites requiring drainage (except those with small amounts of ascites on imaging but asymptomatic);

e. Known presence of symptomatic central nervous system metastases and/or carcinomatous meningitis;

f. History of other primary malignancies, except: 1) malignancies in complete remission for at least 2 years prior to enrolment and requiring no other treatment during the study period; 2) adequately treated non-melanoma skin cancers or malignant freckle like nevus with no evidence of disease recurrence; 3) adequately treated carcinoma in situ with no evidence of disease recurrence;

g. Patients with autoimmune diseases or immunodeficiencies who are treated with immunosuppressive drugs.

h. Patients with bleeding tendency.

i. Pregnant and lactating women.

j. Substance abuse, clinical, psychological, or social factors that would interfere with informed consent or study conduct.

k. Individuals who may be allergic to PD-1 monoclonal antibody, anlotinib, albumin paclitaxel, gemcitabine.

5.3. Criteria for Termination of the Study

Subjects must discontinue the clinical trial if one of the following occurs during the course of the study:

a. Disease progression.

b. The subject expresses unwillingness to continue participation.

c. Serious toxic side effects.

d. Pregnancy.

e. The investigator decides to change the treatment plan based on the patient's condition.

5.4. Withdrawal Criteria

a. Use of other anti-tumor treatments (including chemotherapy, targeted therapy or biologics, etc.) that affect the judgement of efficacy during the study;

b. The occurrence of serious AEs and the investigator judgement is no longer suitable to continue to participate in this study, or unexpected pregnancy;

c. Subjects who are unwilling to continue the clinical trial and insist on proposing withdrawal;

d. The investigator considers it necessary to terminate the study.

6. Determination of Sample Size

The primary endpoint of this single-arm trial was ORR, which was assumed to be 23% based on standard of chemotherapy (AG regimen) as the historical control [5]. It is hypothesized that the incorporation of penpulimab and anlotinib will elevate the ORR to 40% in patients with mPC. With a significance level of 0.05 and a power of 0.8, the required sample size is 59. Accounting for an anticipated dropout rate of 10%, the final required sample size is adjusted to 66. This is determined by a two-sided exact test using PASS 15.0 software.

7. Technical Routes and Processes

Patients with mPC who were identified as treatment-naïve underwent rigorous screening in compliance with the inclusion and exclusion criteria described in the study protocol. After providing informed consent, subjects were administered a combination treatment regimen comprising penpulimab, anlotinib, nab-paclitaxel, and gemcitabine. Select suitable subjects for puncture biopsy of target lesions before treatment, and perform NGS 539-panel gene sequencing of tumor tissue, RNA 289-TME testing if feasible, and peripheral blood circulating tumor DNA (ctDNA) testing according to procedure. The genetic testing and RNA, ctDNA testing during the research process was completed by Jiangsu Simcere Diagnostics Co., Ltd. Patients were followed regularly for safety and efficacy assessments.

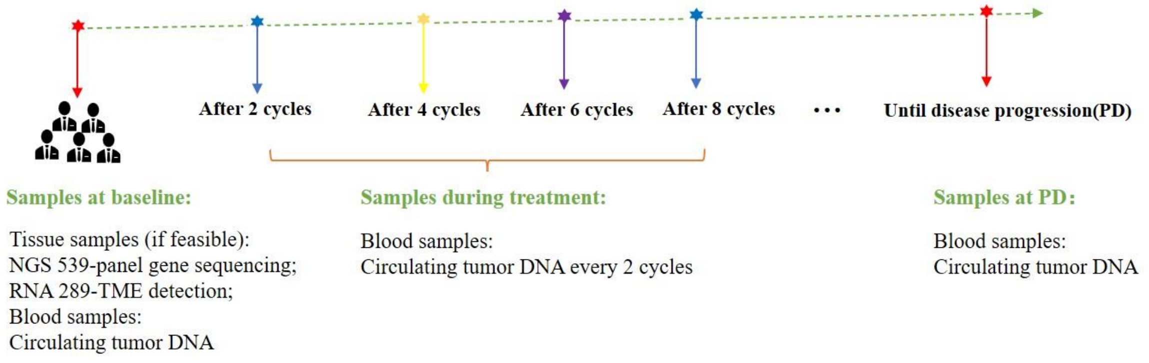

Schematic diagram of the biomarker testing process during treatment

8. Observation Items and Test Time Points

During the study period, the combination therapy will be administered until PD, with each cycle spanning 21 days. AEs will be systematically evaluated following each treatment cycle. The initial efficacy assessment will be performed 2 cycles (6 weeks) after dosing. Subsequent efficacy assessments will be conducted every 2 cycles for the first 8 cycles of treatment (PAAG treatment). If treatment exceeds 8 cycles (24 weeks), efficacy assessments are performed every 3 cycles (9 weeks). Follow-up visits will be conducted every 2 months to observe adverse effects and survival.

Treatment Schedule

Treatment Phase	Screening	Treatment period						Follow-up
Time Point	<14 days before enrollment	After Cycle 1	After Cycle 2	After Cycle 3	After Cycle 4	PAAG treatment, every 2 cycles	Maintenance treatment, every 3 cycles	every 2 months
Medical history (note side effects)	√	√	√	√	√	√	√	√
Physical examination: Height Weight ECOG	√	√	√	√	√	√	√	√
Blood analysis:<br>-Hematology<br>-biochemistry tumor markers<br>-activated lymphocyte count<br>-cytokine determination	√<br>√<br>√<br>√	√<br>√<br>×<br>×	√<br>√<br>√<br>√	√<br>√<br>×<br>×	√<br>√<br>√<br>√	√<br>√<br>√<br>√	√<br>√<br>√<br>√	√<br>√<br>√<br>√
Toxicity assessment	√	√	√	√	√	√	√	√
NGS assay	√							
RNA TME assay	√							
ctDNA	√		√		√	√	√	√
Imaging examination (Chest and abdominal CT/MR/PET/)	√		√		√	√	√	√

9. Observation of Adverse Events

Safety Measures: AEs will be meticulously assessed according to NCI-CTC AE v5.0. Common adverse reactions associated with chemotherapy compass bone marrow suppression, gastrointestinal reactions (such as nausea, loss of appetite), alopecia, fatigue, and abnormal liver function. A small subset of subjects may have allergic phenomena. Recognized adverse effects of PD-1 monoclonal antibody immunotherapy comprise fever, bone pain, joint and muscle soreness, rash or pruritus. A minority of subjects may encounter serious adverse reactions including: immune-related pneumonia, hepatitis, myocarditis, and hypothyroidism, etc. Established adverse effects of antiangiogenic drugs include hypertension, proteinuria, hand-foot skin reactions, diarrhea, thrombosis, and bleeding.

10. Data Safety Monitoring

All AEs must be meticulously documented, appropriately managed and monitored until resolution or stabilization of the condition. Serious AEs and unexpected events should be promptly reported to the Ethics Committee, the competent authority and the drug regulatory authority in a timely manner according to the provisions. The principal investigator will conduct regular cumulative reviews of all adverse events, and convene investigator meetings as needed to assess risks versus benefits. For studies with greater than minimum risk, an independent data monitor will be arranged to monitor the study data. For studies with high risk, an independent data safety monitoring committee will be established for ongoing review of safety and efficacy to determine whether the study should proceed per protocol.

11. General Analysis Considerations

11.1. Data Reporting

Statistical analyses will be presented using summary tables, figures, and data listings.

Individual subject data obtained from the case report forms (CRFs), external laboratory data, and any derived data (such as change from Baseline and percent change from Baseline) will be presented in data listings by subject. Data from all assessments, whether scheduled or unscheduled, will be listed by subject and visit. Unscheduled visits and visits occurring more than one day outside protocol defined window will be excluded from summaries.

All statistical analyses and graphics were managed by computing software R v4.0.2.

11.2. Data Analysis and Summaries

Descriptive statistics will include mean ± standard deviations or median (maximum and minimum) for continuous variables, and frequency (percentage) for categorical variables. 95% confidence intervals will be calculated if necessary.

11.3. Data Handling

11.3.1. Baseline Characteristics

For baseline characteristics, parameters are defined as the most recent no missing values prior to treatment start. No missing value estimation.

11.3.2. Partial Dates

If only a partial date is available and is required for a calculation, the following standards will be applied:

- Date (If the date record is incomplete and does not affect logic)
  - For missing day only: Day will be imputed as the 1st day of the month if does not contradict another date.
  - For missing day and month: Day and month will be imputed as 1st Jan if does not contradict another date.
  - For missing day, month and year: no missing value estimation.
- Efficacy
  - All missing of primary efficacy Measurements due to withdrawal were included in the analysis as "not evaluable”.
  - When calculating the time variables (e.g., PFS, OS), subjects with missing tumor assessment after treatment will be checked on a case-by-case basis to determine the deletion time during data audit.

- Safety
  - No missing value estimation.

11.3.3. Standard Calculations

Variables requiring calculation will be derived using the following formulas:

- **Days**: A duration expressed in days between one date (*date1*) and another later date (*date2*) will be calculated using the following formulas:

duration (days) = date2 - date l + 1

- **Months**: A duration expressed in months is calculated as the number of days divided by 365.25 / 12.
- **Years:** A duration expressed in years between one date (*date1*) and another date (*date2*) is calculated using the following formulas:

duration (years) = (date2 - date1+ 1) / 365.25

- **Age:** Age is calculated as the number of years from the date of birth (*DOB*) to the date of informed consent (*DOIC*). The following formula is used:

age (years) = year of DOIC - year of DOB +1.

12. Analysis Populations

The analysis population includes full analysis set (FAS) and safety set (SS).

12.1. Full Analysis Set (FAS)

The FAS will include the patients who had measurable lesions at baseline, and who have received at least one dose of the study drug. This dataset will be used for the analysis of all efficacy data.

12.2. Safety Set (SS)

The SS will include all subjects who receive any amount of investigational product. Treatment assignment will be based on the treatment actually received. This population will be used for the analysis of all safety data.

13. Study Population

13.1 Subject Disposition

Subject disposition information will be summarized and listed for all subjects. The number and percentage of subjects enrolled, completed or early terminated will be summarized.

13.2. Protocol Deviations

Protocol deviations for missed visits, missed assessments, out of window visits or assessments, and violations of inclusion/exclusion criteria will be determined based on available data. All other protocol deviations will be collected.

13.3. Demographic and Baseline Characteristics

Demographic variables will include the following:

- Age at informed consent
- Sex

Other Baseline characteristics will include the following:

- History of cancer, including disease term, PC location, pathological type, clinical stage, pathological stage, metastatic site, etc.,
- Baseline height and weight (BMI, body surface area)
- Baseline vital signs: systolic blood pressure, diastolic blood pressure, pulse, temperature, respiration, ECOG PS score
- Baseline target and non-target lesions: number of lesions, total diameter, and distribution of lesion sites
- Virus detection and hepatitis B 5 items, HBV DNA, HCV RNA

Demographic and Baseline characteristics will be summarized for the FAS populations. The patients' demographic

characteristics (gender, age), tumor diagnosis information (pathological diagnosis, clinical staging), and other baseline information (height, and weight [body mass index]), vital signs, ECOG PS, and laboratory tests will be analyzed using descriptive statistics.

14. Efficacy Analyses

The primary and secondary efficacy analyses will be based on the FAS Population.

14.1. Primary Efficacy Analyses

Primary efficacy endpoints are ORR and DCR.

14.1.1. ORR

ORR assessed by the investigator: according to RECIST V1.1, the proportion of patients who achieve CR or PR assessed in the analysis population.

14.1.2. DCR

DCR assessed by the investigator: according to RECIST V1.1, the proportion of patients who achieve CR, PR, or SD assessed by the investigator in the analysis population.

14.2. Secondary Efficacy Analyses

Secondary efficacy endpoints are OS and PFS.

14.2.1. OS

OS assessed by the investigator: time from first dose to death recorded for any cause. Patients who are still alive at the time of analysis are censored at the last contacted date. The inter-group comparison of OS will be performed using the stratified log-rank test, the median OS and corresponding 95% CI will be estimated using the Kaplan-Meier method, and the survival curves will be plotted.

14.2.2. PFS

PFS assessed by the investigator: according to RECIST V1.1, the time from first dose to the first recorded imaging disease progression or death caused by any reason as assessed by the investigator, whichever occurs first. Patients who are still alive with no disease progression recorded at the time of analysis are censored at the last imaging evaluation date. Patients who are still alive with no imaging evaluation recorded after baseline are censored at the date of the first dose.

15. Exploratory Analyses

Multi-omics biomarkers associated with clinical response were assessed as an exploratory objective.

16. Safety Analyses

All safety analyses will be based on the Safety Sets, with safety parameters in including AEs, laboratory tests, vital signs, 12-lead ECG test, etc.

16.1. Adverse Events (AEs)

All AEs will be coded and classified using Medical Dictionary for Regulatory Activities (MedDRA), and graded as per Common Terminology Criteria for Adverse Events (CTCAE) V5.0.

All AE summaries will be restricted to treatment-emergent adverse events (TEAEs), which are defined as any AEs that newly appear, increase in frequency, or worsen in severity following initiation of study medication. The incidences (frequency) of all TEAEs, TEAEs at grade 3 and above, TEAEs related to the study drugs, immune-related AEs (irAEs), serious AEs (SAEs), SAEs related to study drugs, TEAEs leading to discontinued study medication, and TEAEs leading to study termination will be summarized, and the above-mentioned AEs will be summarized based on System Organ Classes (SOCs) and Preferred Terms (PTs) in MedDRA coding. In addition, the severity levels of TEAEs and relationship with the study drug were also summarized by SOCs and PTs.

The following listings will be presented by subject:

- All AEs
- Serious AEs (subset of the AEs where serious is marked as "Yes")
- Death information will be provided in a separate listing, should any deaths occur
- Severe AEs (subset of AEs where severity is marked as "Severe" or severity is missing)
- Related AEs (subset of AEs where relationship to study medication is marked as "Definite", "Possible" or "Probable")
- AE's leading to withdrawal of investigational product (subset of AEs where action taken with study medication is marked as "Drug Withdrawn")
- AE's leading to Study Discontinuation (subset of AEs where subject discontinued from study is checked)

16.2. Clinical Laboratory Evaluations

A listing of available laboratory reference/normal ranges for each laboratory parameter will be provided including age, sex, values with units. For laboratory tests, the observed values and changes from the baseline will be analyzed using descriptive statistics. The baseline results and the worst results during the trial were presented in a crosstab. Laboratory test abnormalities will be graded and summarized according to CTCATE V5.0.

16.3. Vital Signs, physical examinations, and other safety-related examinations

Measured values and changes from baseline for vital signs, physical examinations, and other safety-related examination values will be analyzed using descriptive statistics. The baseline results and the worst results during the trial were presented in a crosstab.

ECOG PS will be analyzed and summarized using descriptive statistics.

16.4. 12-lead Electrocardiograms (ECGs) test

Descriptive statistics are used for 12-lead ECG and changes from baseline. A cross-classification table is used to describe ECG and changes from baseline before and after the treatment and data lists will be provided.

17. Ethics of clinical study

The clinical study will follow the relevant provisions of Declaration of Helsinki developed by WMA. The clinical study was not conducted until the trial protocol was approved by the ethics committee before the start of the study. Before each subject is enrolled in this study, the investigator is responsible for introducing the purpose, procedures and possible risks of this study to the subject or his/her representative in a complete and comprehensive manner, and signing the written informed consent form. The subjects should be informed that they have the right to withdraw from this study at any time. The informed consent form is retained as a clinical study document for future reference. Personal privacy and data confidentiality of subjects will be protected during the study.

18. Study Schedule

April 2022-December 2023: Screening, treatment, follow-up;

December 2023-December 2024: Follow-up visit, data collection and statistics;

19. References

1. Siegel RL, Miller KD, Jemal A. Cancer statistics, 2022. CA Cancer J Clin. 2022 Jan;72(1):7-33.

2. Chen WQ, Li H, Sun KX, et al. [Report of Cancer Incidence and Mortality in China, 2014]. Zhonghua Zhong Liu Za Zhi. 2018 Jan 23;40(1):5-13.

3. Quante AS, Ming C, Rottmann M, et al. Projections of cancer incidence and cancer-related deaths in Germany by 2020 and 2030. Cancer Med. 2016 Sep;5(9):2649-56.

4. Kamisawa T, Wood LD, Itoi T, et al: Pancreatic cancer. Lancet. 2016 Jul 2;388(10039):73- 85.

5. Von Hoff DD, Ervin T, Arena FP, et al. Increased Survival in Pancreatic Cancer with nab- Paclitaxel plus Gemcitabine.

N Engl J Med. 2013 Oct 31;369(18):1691-703.
6. Gourgou-Bourgade S, Bascoul-Mollevi C, Desseigne F, et al. Impact of FOLFIRINOX Compared With Gemcitabine on Quality of Life in Patients With Metastatic Pancreatic Cancer: Results From the PRODIGE 4/ACCORD 11 Randomized Trial. J Clin Oncol. 2013 Jan 1;31(1):23-9.
7. Tempero MA, Malafa MP, Al-Hawary M, et al. Pancreatic Adenocarcinoma, Version 2.2021, NCCN Clinical Practice Guidelines in Oncology. J Natl Compr Canc Netw. 2021 Apr 1;19(4):439-457.
8. O'Donnell JS, Teng MWL, Smyth MJ. Cancer immunoediting and resistance to T cell- based immunotherapy. Nat Rev Clin Oncol. 2019 Mar;16(3):151-167.
9. Pan C, Liu H, Robins E, Song W, Liu D, Li Z, Zheng L. Next-generation immuno- oncology agents: current momentum shifts in cancer immunotherapy. J Hematol Oncol. 2020 Apr 3;13(1):29.
10. Zhang M, Sun H, Zhao S, et al． Expression of PD-L1 and prognosis in breast cancer: a meta-analysis. Oncotarget. 2017 May 9;8(19):31347-31354.
11. Palmeri M, Mehnert J, Silk AW, et al． Real-world application of tumor mutational burden-high (TMB-high) and microsatellite instability (MSI) confirms their utility as immunotherapy biomarkers. ESMO Open. 2022 Feb;7(1):100336.
12. O'Hara MH, O'Reilly EM, Varadhachary G, et al. CD40 agonistic monoclonal antibody APX005M (sotigalimab) and chemotherapy, with or without nivolumab, for the treatment of metastatic pancreatic adenocarcinoma: an open-label, multicentre, phase 1b study. Lancet Oncol. 2021 Jan;22(1):118-131
13. Henriksen A, Dyhl-Polk A, Chen I, et al. Checkpoint inhibitors in pancreatic cancer. Cancer Treat Rev. 2019; 78:17-30.
14. Dhillon S. Penpulimab: First Approval. Drugs. 2021 Dec;81(18):2159-2166.
15. Zheng Y, Mislang ARA, Coward J, et al. Penpulimab, an anti-PD1 IgG1 antibody in the treatment of advanced or metastatic upper gastrointestinal cancers. Cancer Immunol Immunother. 2022 Feb 15. doi: 10.1007/s00262-022-03160-1.
16. Vasudev NS, Reynolds AR. Anti-angiogenic therapy for cancer: current progress, unresolved questions and future directions. Angiogenesis. 2014 Jul;17(3):471-94.
17. Moore MJ，Goldstein D，Hamm J，et al． Erlotinib plus gemcitabine compared with gemcitabine alone in patients with advanced pancreatic cancer: A phase III trial of the national cancer institute of canada clinical trials group． J Clin Oncol, 2007, 25(15): 1960-1966．
18. Kindler HL， Niedzwiecki D， Hollis D， et al． Gemcitabine plus bevacizumab compared with gemcitabine plus placebo in patients with advanced pancreatic cancer: Phase III trial of the cancer and leukemia group b (calgb 80303)． J Clin Oncol,2010,28 (22):3617- 3622．
19. Van Cutsem E, Vervenne WL, Bennouna J, Humblet Y, Gill S, Van Laethem JL, Verslype C, Scheithauer W, Shang A, Cosaert J, Moore MJ. Phase III trial of bevacizumab in combination with gemcitabine and erlotinib in patients with metastatic pancreatic cancer. J Clin Oncol. 2009 May 1;27(13):2231-7.
20. Watkins DJ, Starling N, Cunningham D, Thomas J, Webb J, Brown G, Barbachano Y, Oates J, Chau I. The combination of a chemotherapy doublet (gemcitabine and capecitabine) with a biological doublet (bevacizumab and erlotinib) in patients with advanced pancreatic adenocarcinoma. The results of a phase I/II study. Eur J Cancer. 2014 May;50(8):1422-9.
21. Song Y, Fu Y, Xie Q, Zhu B, Wang J, Zhang B. Anti-angiogenic Agents in Combination with Immune Checkpoint Inhibitors: A Promising Strategy for Cancer Treatment. Front Immunol. 2020 Aug 25; 11:1956.
22. Shen G, Zheng F, Ren D, Du F, Dong Q, Wang Z, Zhao F, Ahmad R, Zhao J. Anlotinib: a novel multi-targeting tyrosine kinase inhibitor in clinical development. J Hematol Oncol. 2018;11(1):120.
23. Si X, Zhang L, Wang H, Zhang X, Wang M, Han B, Li K, Wang Q, Shi J, Wang Z, Cheng Y, Shi Y, Chen W, Wang X, Luo Y, Nan K, Jin F. Management of anlotinib-related adverse events in patients with advanced non-small cell lung

cancer: Experiences in ALTER-0303. Thorac Cancer. 2019 Mar;10(3):551-556.

24. Li S. Anlotinib: A Novel Targeted Drug for Bone and Soft Tissue Sarcoma. Front Oncol. 2021 May 20; 11:664853.
